# A review of the *Paectes arcigera* species complex (Guenée) (Lepidoptera, Euteliidae)

**DOI:** 10.3897/zookeys.264.3274

**Published:** 2013-02-06

**Authors:** Michael G. Pogue

**Affiliations:** 1 Systematic Entomology Laboratory, PSI, Agricultural Research Service, U. S. Department of Agriculture, c/o Smithsonian Institution, P.O. Box 37012, NMNH, MRC-168, Washington, DC 20013-7012, USA

**Keywords:** Taxonomy, new species, Brazilian peppertree, *Schinus terebinthifolius*, Anacardiaceae, invasive species, new host records

## Abstract

Five new species of *Paectes* Hübner [1818] related to *Paectes arcigera* (Guenée) (Puerto Rico, U.S. Virgin Islands, British Virgin Islands, Guadeloupe, Dominica, St. Lucia, Trinidad) and *Paectes longiformis* Pogue (Brazil) are described: *Paectes asper* **sp. n.** (Florida, Bahamas, Cuba, Cayman Islands, Jamaica, Haiti, Dominican Republic, Puerto Rico, British Virgin Islands, U.S. Virgin Islands, Dominica, Colombia), *Paectes medialba* **sp. n.** (Argentina), *Paectes similis* **sp. n.** (Brazil), *Paectes sinuosa* **sp. n.** (Argentina, Brazil, Paraguay), and *Paectes tumida* **sp. n.** (Colombia, Guyana, Suriname, French Guiana). Adults and genitalia are illustrated for all species. Taxonomic changes include the **rev. stat.** of *Paectes nana* (Walker) (Florida, Greater Antilles, Mexico, Guatemala, Galapagos) as a valid species and **revised synonyms** *Paectes indefatigabilis* Schaus and *Paectes isabel* Schaus as junior synonyms of *Paectes nana* instead of *Paectes arcigera*. New host records for *Paectes sinuosa* and *Paectes nana* reared on Brazilian peppertree (*Schinus terebinthifolius* Raddi, Anacardiaceae) are presented. The holotype and female genitalia of *Paectes obrotunda* (Guenée) are illustrated.

## Introduction

Specimens of a species described as *Paectes longiformis* Pogue were sent to me for identification from scientists at the Biological Control Research and Containment Laboratory, University of Florida, Ft. Pierce, FL. This species is being tested for possible release as a biological control agent of the Brazilian peppertree (*Schinus terebinthifolius* Raddi, Anacardiaceae), an invasive species with severe economic impact. Specimens originated near the airport in Salvador, Bahia, Brazil. Originally thought to be *Paectes obrotunda* (Guenée), it proved to be a new species (Manrique et al. 2012).

In the collection of the USNM there were over 250 specimens identified as *Paectes obrotunda*. The results of this study showed that these specimens consisted of two described species, *Paectes arcigera* (Guenée) and *Paectes nana* (Walker) and five additional new species that are described here. Taxonomic changes included the revised status of *Paectes nana* as a valid species and not a synonym of *Paectes arcigera*. *Paectes burserae* (Dyar) is a syn. n. of *Paectes nana*. *Paectes indefatigabilis* Schaus and *Paectes isabel* Schaus, both from the Galapagos Islands, Ecuador, are synonyms of *Paectes nana* and not *Paectes arcigera* as previously thought (Poole 1989; Roque-Álbelo and Landry 2011). *Paectes obrotunda* (Guenée) is also referred to the *Paectes arcigera* group.

The *Paectes arcigera* group includes only the species referred to in this paper. Species in this group can be recognized by the elongate free saccular extension in the male genitalia. Including the species in this revision there are 12 species of *Paectes* in North America and 40 species in the Neotropics. Two of these species, *Paectes nana* and *Paectes asper* Pogue, occur both in North America and the Neotropics.

## Material and methods

### Repository abbreviations

Specimens and images were examined from the following collections:

BMNH The Natural History Museum, London, UK

LAN Peter J. Landolt collection, Yakima, WA, USA

MGCL McGuire Center for Lepidoptera and Biodiversity, University of Florida, Gainesville, FL, USA

TDC Terhune S. Dickel Collection, Ocala, FL, USA

UFPC Coleção Entomológica Padre Jesus Santiago Moure, Universidade Federal do Paraná, Curitiba, BRAZIL

USNM National Museum of Natural History, Washington, DC, USA

WSU Washington State University, Pullman, WA, USA

Dissection of genitalia follows the method of Pogue (2002) except specimens were mounted in Euparal and stained exclusively in Mercurochrome. Male genital morphology follows Forbes (1954) and female morphology follows Lafontaine (2004). Terms used in describing forewing morphology follow Lafontaine (2004). Images of adult moths were taken with a Visionary Digital Imaging System using a Canon EOS 5D Mark II camera with a modified K2 long-distance lens and a pulsed xenon flash. Forewing length was measured using a calibrated ocular micrometer from the juncture of the thorax to the apex, including fringe.

Distribution maps (Figs 48–52) were generated using ESRI^®^ ArcMap^™^ 10.0 (ESRI, Redland, CA). Latitude and longitude coordinates were obtained from the label data or from a localities database that I maintain. The data points were entered into a FileMaker Pro 11.0 v 3 database and then directly assembled as a data layer onto a world map projection using a GCS-WGS-1984 Geographic Coordinate System.

### Key to species based on male genitalia

**Table:** 

1	Free saccular extension extending above costa (Fig. 29)	2
–	Free saccular extension extending below costa (Fig. 31)	6
2	Free saccular extension wide, apex enlarged (Fig. 29)	*Paectes arcigera*
–	Free saccular extension narrow, apex not enlarged (Fig. 30)	3
3	Setae on dorsal surface of valve hairlike, straight (Fig. 30)	*Paectes longiformis*
–	Setae on dorsal surface of valve thick, curved (Fig. 32)	4
4	Lateral margin of valve bearing wide, flat setae on sclerotized ridge (Fig. 32)	*Paectes nana*
–	Lateral margin of valve lacking wide flat setae	5
5	Free saccular extension sinuate; base covered with minute spicules (Fig. 35)	*Paectes sinuosa*
–	Free saccular extension straight, curved near apex; base lacking minute spicules (Fig. 33)	*Paectes asper*
6	Setae on dorsal surface of valve hairlike, straight; free saccular extension lacking spicules (Fig. 31)	*Paectes similis*
–	Setae on dorsal surface of valve thick, curved; free saccular extension covered with minute spicules	7
7	Base of free saccular extension bulbous, more than twice width of arm below apex (Fig. 36)	*Paectes tumida*
–	Base of free saccular extension gradually narrowing toward apex, not bulbous (Fig. 34)	*Paectes medialba*

### Key to species based on female genitalia

**Table:** 

1	Lateral margin of 8th sternite produced into short, triangular projections (Fig. 40)	2
–	Lateral margin of 8th sternite smooth, lacking projections (Fig. 37)	6
2	Ductus bursae at juncture with appendix bursae approximately same width as juncture with corpus bursae (Fig. 38)	3
–	Ductus bursae at juncture with appendix bursae narrow at juncture with appendix bursae and widens at juncture with corpus bursae (Fig. 41)	*Paectes medialba*
3	Ostium bursae with a medial, curved, sclerotized bar (Fig. 43)	*Paectes tumida*
–	Ostium bursae without an obvious sclerotized structure (Fig. 38)	4
4	Lateral margin of 8th sternite not well developed, apex pointing laterally (Fig. 38)	*Paectes longiformis*
–	Lateral margin of 8th sternite well developed, apex pointing ventrally (Fig. 40)	5
5	Juncture of appendix bursae and ductus bursae just distal to ostium bursae (Fig. 40)	*Paectes asper*
–	Juncture of appendix bursae at middle of ductus bursae (Fig. 42)	*Paectes sinuosa*
6	Ostium bursae a round circle (Fig. 39)	*Paectes nana*
–	Ostium bursae a sclerotized band or half-circle	7
7	Ostium bursae a large, heavily sclerotized half-circle shape (Fig. 37)	*Paectes arcigera*
–	Ostium bursae a sclerotized band with narrowed lateral apices (Fig. 46)	*Paectes obrotunda*

## Descriptions

### Paectes arcigera

(Guenée, 1852)

http://species-id.net/wiki/Paectes_arcigera

Figs 1–4 29 37 48

Ingura arcigera Guenée *in*Boisduval and Guenée 1852: 312.

#### Type material.

St. Thomas: lost. **Neotype:** Dominica. USNM, here designated. This is a confusing group of species that can only be identified reliably by genitalic characters, so to ensure the stability of the name, a male labeled “DOMINICA: Grande Savane, 1 July 1964, O. S. Flint, Jr., genitalia slide male, USNM 135918 [green label]” is designated as neotype for *Ingura arcigera*Guenée, 1852.

#### Other material examined. 

All specimens in USNM unless noted (62 males, 49 females). **BRITISH VIRGIN ISLANDS:** Guana Island, 1–14 July 1984 (22 males, 11 females), Genitalia slides m, USNM 135957, 1359980, 135991, 135993, 136010, S.E. & P.M. Miller; Virgin Gorda Island, Virgin Gorda Peak, ca. 400 m, 17–19 July 1986 (4 males, 1 female), Genitalia slide m, USNM 135958, S.E. Miller & M.G. Pogue. **DOMINICA:** same data as neotype (1 male, 1 female), genitalia slide male, USNM 136004, 13 May 1964 (1 male), 14 June 1964 (1 male), 28 Oct. 1966 (2 males), E.L. Todd, 31 Oct. 1966 (2 males), genitalia USNM 136003, E.L. Todd, 1 Nov. 1966 (1male), E.L. Todd; Clarke Hall, 11 Jan. 1965 (1 female), J. F. G. Clarke & Thelma M. Clarke, 16 Jan. 1965 (1 male), J. F. G. Clarke & Thelma M. Clarke; 2.2 mi E of Pont Casse, 7 May 1964, O. S. Flint, Jr. (1 male); Roseau, Nov. 1967 (1 female), N.L.H. Krauss; S. Chiltern (1 female), 8–10 Dec. 1964 (1 female), P.J. Spangler; no specific locality, May-June 1905 (1 male, 4 females), Genitalia slide m MGP 1325, E. A. Agar [BMNH], Oct. 1904 (1 male, 3 females), Nov. 1904 (1 female), Apr. 1905 (1 male), E. A. Agar [BMNH], (2 males, 2 females), Genitalia slide m MGP 1324, E. A. Agar [BMNH], (2 males, 6 females) [BMNH]; Portsmouth, 8 Oct. 1956 (1 female), E. Hamblett [BMNH]. **GRENADA:** St. George’s Cave, July 18 (1 male, 1 female), genitalia slide male MGP 1321 [BMNH]. **GRENADINES:** Union I., June 1905 (1 male), genitalia slide MGP 1322 [BMNH]. **GUADELOUPE:** Port de Jaray, 14 Sep. 1982 (1 male), B. Lalanne-Cassou. **PUERTO RICO:** Bayamón, 15 Jan. 1933 (1 female), Anderson & Lesesny; Guanica, Fajardo, 29 July 1913 (1 male), E. G. S. Collector; Maricao, Centro Vacacional, Monte del Estado, nr. Maricao, 1–9 Mar. 1971 (1 male), C.P. Kimball; Puerto Rico, Mayaguez, 3–4 Aug. 1955 (1 female), J.A. Ramos; San Juan, June–July 1932 (1 male), Genitalia slide USNM 135929, C.G. Anderson. **ST. LUCIA:** no specific locality, (2 males, 4 females), Branch; (4 males, 3 females), Maj. Cowrie, (2 males, 1 female) [BMNH]; 1 mi NW Soufriere, 18–23 Nov. 1975 (1 male), Genitalia slide USNM 135933, E.L. Todd. **ST. VINCENT:** Bequia I., Sep. 1903 (2 females); windward side, (1 male), H. H. Smith [BMNH]. **TRINIDAD:** No specific locality (1 female), A. Busck. **U. S. VIRGIN ISLANDS:** ST. CROIX: 1 mi W airport, 6–16 July 1967 (1 male), Genitalia slide USNM 42808; Christiansted, 19 Nov. 1941 (1 male), H.A. Beatty; Gallows Point, 9 July 1956 (1 female), genitalia slide USNM 136045, J.G. Coutsis; Orangegrove, W. End, 6–16 July 1967 (1 male), E.L. Todd.

#### Diagnosis.

The only reliable way to distinguish *Paectes arcigera* from *Paectes asper* Pogue is by characters in the male and female genitalia. Male genitalia of *Paectes arcigera* consist of a reduced, fingerlike valve and costa, and a greatly expanded free saccular extension (Fig. 29). In *Paectes asper* the valve is triangulate, the costa has a truncate apex, and the free saccular extension (Fig. 33) is approximately half the width as in *Paectes arcigera*. Female genitalia of *Paectes arcigera* have a large, half-round ostium bursae covered with thorn-like spines and the lateral apices of the eighth sternite are not produced (Fig. 37). In *Paectes asper*, the ostium bursae is a crescent-shaped invagination covered with fine spicules and the lateral apices of the eighth sternite are produced (Fig. 40).

#### Redescription.

**Adults.** Sexes dimorphic. **Male.** *Head* – antenna broadly bipectinate to 3/5 length, then filiform; eyes large, globular; vertex with broad scales, cream colored, thin black lines adjacent to scape; frons with broad scales, projecting slightly beyond anterior eye margin, mostly cream colored with a few gray and ferruginous scales, two black dots along eye margin, one ventral to antenna, other dorsal to palp; labial palp porrect, mixture of cream-colored, gray, and ferruginous scales, internal surface white. *Thorax* – prothorax somewhat variable, well-marked specimens cream colored with medial ferruginous band, anterior margin a thin black line, posterior margin gray to black and not as well defined as anterior line; patagium with cream-colored hairlike scales mixed with ferruginous, gray, and black scales; protibia cream colored mixed with a few black scales; tarsi gray with white apical bands; middle legs mixture of cream-colored and pale gray scales, scales much longer than either the pro- or hind tibia, tarsi pale gray with cream-colored apical bands; hind tibia cream colored, tarsi cream colored; underside with white hairlike scales; forewing length 9.5–12.2 mm; costal area a gray; ovate basal spot white in ventral half, pale gray white-tipped scales in dorsal half; antemedial line thin, black, from posterior margin to Cu vein, forming ventral border of basal spot; medial area between antemedial and postmedial lines mostly white mixed with pale-gray scales; some specimens with medial line consisting of two very thin crescent-shaped lines from posterior margin to just below M3 vein; reniform spot obscure, consists of two small ferruginous dots in a vertical pattern, or may only be represented by a single dot in some specimens; postmedial line a mixture of ferruginous and black scales, double line from posterior margin to vein M2 then single until merging with black dash at vein R5 that extends to outer margin; white apical spot; subterminal area variable, gray to cream colored, mixed with ferruginous scales; terminal line a series of black dashes between veins; fringe light brown to gray with gray patches at wing veins resulting in a somewhat checkered appearance; hind wing with marginal shading dark gray, veins highlighted dark gray, white between veins and at base, anal fold a white and dark gray striped pattern. *Abdomen* – variable, mixture of cream-colored, light-brown, and ferruginous scales, posterior margin of dorsal segments with short, black line; venter variable, can be white with faint, black medial stripe to a pair of wide black or brown stripes with a thin central line of the same color on a white background; male eighth segment membranous with a pair of short, sternal, sclerotized bars and a pair of longer, wider, dorsal sclerotized bars; a pair of lateral, coremata bearing numerous, fine, elongate setae. *Genitalia* (Fig. 29) – Uncus triangulate, apex recurved and pointed, 0.62–0.64 × length of subscaphium; subscaphium triangulate, decurved, pointed apex; valve membranous, reduced, widest at base and tapering to a fingerlike projection, setose; costal margin sinuate, apex produced, fingerlike projection wider than valve, setose; sacculus well developed, proximal half fused with valve, distal half free, elongate, broad, curved inward, longer than valve, apex broadly rounded; saccus U-shaped; aedeagus straight, slightly curved in distal half, dorsum near apex covered with minute spicules; vesica irregularly-shaped oval, wide diverticulum lateral to apex of aedeagus, a long, flat cornutus at base of vesica directed posteriorly, short, thumb-like diverticulum near apex and adjacent to irregular sclerotized area bearing a short, narrow cornutus. **Female.** As in male except:antenna filiform; forewing length 9.8–11.6 mm;medial area less contrasting with fewer white scales than male; overall more drab in appearance than male. *Genitalia* (Fig. 37) – Papillae anales ovate, soft, fleshy, covered with numerous setae; anterior apophyses fused with eighth segment; posterior apophyses present; venter of eighth segment covered with minute spicules; ostium bursae sclerotized, rectangulate, dorsal invagination covered with thorn-like spines, dorsal spines largest; base of ductus bursae rectangulate and fused with eighth segment; ductus bursae juncture with appendix bursae below base, striate; duct of appendix bursae narrow, striate 2/3 length; appendix bursae membranous, round; corpus bursae ovate to round, covered internally with numerous thorn-like signa.

**Figures 1–8.**
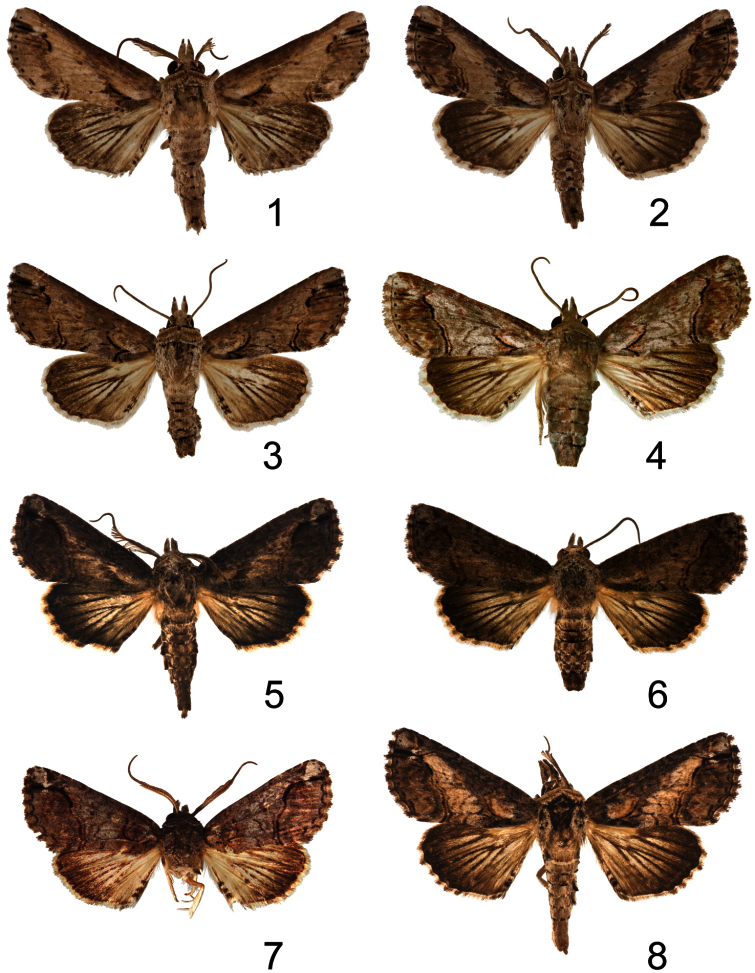
*Paectes* adults. **1** *Paectes arcigera* ♂, Virgin Gorda Peak, Virgin Gorda Island, British Virgin Islands, 17–19 July 1986, S. E. Miller & M. G. Pogue **2** *Paectes arcigera* ♂, Grand Savane, Dominica, 1 July 1964, O. S. Flint, Jr. **3** *Paectes arcigera* ♀, Grand Savane, Dominica, 1 July 1964, O. S. Flint, Jr. **4** *Paectes arcigera* , Guana Island, British Virgin Islands, 1–14 July 1984, S. E. & P. M. Miller **5** *Paectes longiformis* ♂, Holotype, nr. Salvador Airport, Bahia, Brazil, March 2010, R. Diaz, V. Manrique & M. Vitorino **6** *Paectes longiformis* ♀, nr. Salvador Airport, Bahia, Brazil, March 2010, R. Diaz, V. Manrique & M. Vitorino **7** *Paectes similis* ♂, Holotype, Pernambuco [Recife], Pernambuco, Brazil, Pickel Coll. **8** *Paectes nana* ♂, nr. San Vicente, Hidalgo, Mexico, 2 July 1965, Flint & Ortiz.

#### Distribution and biology

**.** *Paectes arcigera* is restricted to the eastern Caribbean Islands from Puerto Rico and the Lesser Antilles, including U.S. Virgin Islands, British Virgin Islands, Guadeloupe, Dominica, St. Lucia, and Trinidad (Fig. 48). Probably flies throughout the year with flight records for all months except February. Nothing is known about biology or host plants. McMullen (1986) remarked that adults of *Paectes arcigera* were seen near *Crytocarpus pyriformis* Kunth (Nyctaginaceae) on Isla Santa Fe, Galapagos Islands, but this record can be referred to *Paectes nana*.

#### Remarks.

*Paectes arcigera* has been confused in collections and in the literature as *Paectes obrotunda*. Kimball (1965) and Minno (1992) listed *Paectes arcigera* as occurring in Florida, but these are based on a broad concept of *Paectes arcigera* that made this revision of the species complex necessary. These Florida records are now known to be referable to *Paectes asper* and *Paectes nana*. Franclemont and Todd (1983) and Lafontaine and Schmidt (2010) also listed *Paectes arcigera* as occurring in North America, unaware that the name represented a species complex. Askew (1994) listed *Paectes arcigera* as occurring on Little Cayman Island, but this is probably referable to *Paectes asper*. *Paectes arcigera* and *Paectes asper* occur sympatrically in the U.S. Virgin Islands, British Virgin Islands, and Dominica.

### Paectes longiformis

Pogue, 2012

http://species-id.net/wiki/Paectes_longiformis

Figs 5–6 30 38 49

Paectes longiformis Pogue *in*Manrique et al. 2012: 167.

#### Type material.

**Holotype** male – **BRAZIL**: Bahia, nr. Salvador airport (12.91007°S, 38.3380°W), March 2010, R. Diaz, V. Manrique, M. Vitorino; USNM ENT 00148675; HOLOTYPE / *Paectes* *longiformis* Pogue” [red label]. UFPC. Paratypes – (29 males, 30 females) Same data as holotype; genitalia slide male USNM 134921; genitalia slides female USNM 135919, 135976, 135920, 135015, 135016. UFPC, USNM, CNC, BMNH.

#### Diagnosis.

*Paectes longiformis* is most likely to be confused with *Paectes similis* Pogue, but can be differentiated by the color of the medial area of the forewing. In *Paectes longiformis* (Figs 5–6), the medial area is cream colored interspersed with some dark-ferruginous scales and in *Paectes similis* (Fig. 7) this area is shiny white with some gray scales. The tarsi in *Paectes longiformis* are dark gray, whereas in *Paectes similis* they are mostly white with some gray scales. There are several differences in the male genitalia to differentiate *Paectes longiformis* from *Paectes similis*. The uncus is 0.62–0.70 × the length of the subscaphium in *Paectes longiformis* (Fig. 30) and 0.80 × in *Paectes similis* (Fig. 31). The distal, free saccular extension is longer than the valve in *Paectes longiformis* (Fig. 30) and shorter than the valve in *Paectes similis* (Fig. 31). The female genitalia are easily separated from the other species in this group by the form of the lateral margins of the eighth sternite. In *Paectes longiformis*,the lateral margins of the eighth sternite are slightly produced and form a right angle (Fig. 38). In *Paectes arcigera* and *Paectes nana*, the lateral margins are not evident (Figs 37, 39) and in *Paectes asper*, *Paectes medialba* Pogue, and *Paectes sinuosa* Pogue, the lateral margins are produced (Figs 40–42).

#### Distribution and biology.

Known only from the type locality in northeastern Brazil in the state of Bahia (Fig. 49). Larvae have been reared from the Brazilian peppertree. It is possible that this species has a much broader range, considering the wide range of its host plant, but so far no other specimens have been collected.

#### Remarks.

As a potential biological control agent against the Brazilian peppertree in Florida, baseline data was developed about the biology and temperature requirements of *Paectes longiformis* (Manrique et al. 2012).

### Paectes similis

Pogue sp. n.

urn:lsid:zoobank.org:act:054BD467-B4DA-4B4A-A923-E54FB873577E

http://species-id.net/wiki/Paectes_similis

Figs 7 31 49

#### Type material.

**Holotype** male – **BRAZIL:** Pernambuco [Recife], Pickel Coll. Genitalia slide, USNM 135911 [green label]; HOLOTYPE / *Paectes* *similis* Pogue” [red label]. USNM.

#### Etymology.

The species name is the Latin term for “like” which refers to the similarity between this species and *Paectes longiformis*.

#### Diagnosis. 

Comparison of *Paectes similis* to *Paectes longiformis* was given above.

#### Description.

**Adult.** **Male.** FW length 10.5 mm. *Head* – vertex with broad scales, pale gray; labial palp porrect, mixture of pale-gray, ferruginous, and brown scales tipped white, internal surface white; eyes large, globular; frons with broad scales, pale gray, dark brown scales at margin of vertex; male antenna broadly bipectinate to just beyond half length then filiform. *Thorax* – prothorax pale gray, anterior margin with thin black line; patagium pale-gray scales tipped white, a few dark-brown scales anteriorly, mixed with hairlike scales; protibia mixed with white and brown scales, anterior margin white, tarsi brown with white apical bands; midtibia mostly white with some light-brown scales medially, tarsi white; hind tibia white, tarsi white; forewing costal area with pale-gray scales tipped white, minute white dots from postmedial line to apex; prominent white apical spot; basal area ferruginous mixed with a few pale-gray tipped white and white scales; antemedial line black, incomplete, from anal vein to R vein, forming distal border to basal area; medial area slightly paler in overall color from terminal area, consisting of pale-gray and white scales; reniform spot obscure, a pair of tiny dark-brown dots, ventral dot just above M vein in discal cell and larger than dorsal dot, which consists of only a few dark scales; postmedial line black outlined with some ferruginous scales, a double line from posterior margin to vein CuA2, continuing as single line to connecting point with black dash between veins R5 and M1 that extends to outer margin; subterminal area a mixture of light-brown, gray, white, and ferruginous scales, darker than medial area; terminal line a series of black, recurved lines between veins; fringe dark gray at veins, pale gray between veins giving checkered appearance; hind wing white, marginal shading and veins highlighted dark gray, anal fold diffuse white and dark-gray striped pattern. *Abdomen* – eighth segment membranous with pair of dorsal sclerotized bars; pair of lateral coremata bearing numerous, fine, elongate setae. *Genitalia* (Fig. 31) – Uncus triangulate, apex recurved and pointed, 0.8 × length of subscaphium; subscaphium triangulate, decurved, apex pointed; valve membranous, rectangulate, apex truncate, ventrally covered with elongate setae; costal margin sinuate, produced into a truncate lobe, apex round, elongate setae dorsally from middle to apex; sacculus well developed, proximal half fused with valve, distal half free, curved toward midline, apex narrowly rounded; saccus elongate, V-shaped; aedeagus slightly decurved, dorsally spiculate in distal half; base of vesica slightly wider than apex of aedeagus, spiculate ventrally with 2 leaf-like ventral cornuti, becomes ovate with lateral, slightly curved diverticulum, opposite lateral diverticulum a sclerotized, spiculate, grooved area with very short cornutus, two bump-like diverticula on either side of grooved area. **Female.** Unknown.

#### Distribution.

Known only from the type locality (Fig. 49).

#### Remarks.

The holotype of *Paectes similis* was in a series of specimens in the USNM collection identified as *Paectes obrotunda*. Though known only from the type specimen, it is described so as to help eliminate confusion in this complex group of look-a-like species.

### Paectes nana

(Walker) stat. rev.

http://species-id.net/wiki/Paectes_nana

Figs 9–11 32 39 50

Edema nana Walker, 1865: 425.
Ingura burserae Dyar, 1901: 455. **syn. n.**
Paectes indefatigabilis Schaus, 1923: 38. **syn. rev.** (previously synonymized by Poole 1993 under *Paectes arcigera*)
Paectes isabel Schaus, 1923: 39. **syn. rev.** (previously synonymized by Poole 1993 under *Paectes arcigera*)

#### Type material.

*Edema nana* – Type locality: “Dominican Republic, Santo Domingo” Holotype male. UMO; photograph examined.

*Ingura burserae* – Type locality: USA: Florida, Palm Beach. Syntypes male, female. USNM; types examined. Dyar listed two types, a male and female, in his original description. I hereby designate the male as lectotypeto avoid confusion in this complicated group.

*Paectes indefatigabilis* – Type locality: [Ecuador]: [Galapagos Islands]: Indefatigable, Conway Bay. Lectotype male. USNM; examined. Todd (1973) designated the lectotype.

*Paectes isabel* – Type locality: [Ecuador]: [Galapagos Islands]: Indefatigable, Conway Bay. Holotype male. USNM; examined.

#### Other material examined.

All from USNM unless noted. (121 males, 91 females). **COLOMBIA:** BOYACA: Muzo, 400–800 m, (1 female), Fassl [BMNH]. CAUCA: Popayan, May 1972 (1 male), R. Perry [BMNB]. MAGDALENA: Don Amo, 2000 ft., June 1911 (6 males, 1 female), genitalia slide male MGP 1302, female MGP 1304, 4000 ft., (2 males, 1 female), H. H. Smith [BMNH]; Minca, 2000 ft., (2 males), June (1 male, 1 female), H. H. Smith [BMNH]; Valparaiso, 4000 ft., (2 males, 1 female), genitalia slide male MGP 1300, H. H. Smith [BMNH]. SANTA MARTA: Onaca, June–Aug. (2 males, 6 females), genitalia slide male MGP 1301, female MGP 1303, C. Engelke [BMNH]. **COSTA RICA:** no specific locality, (1 male) genitalia slide MGP 1329, Underwood, BMNH; GUANACASTE: Area de Conservacion Guanacaste, Mundo Nuevo, Quebrada Tibio Perla, 300 m, 26 Nov. 2009 (1 female), J. Cortez, host: *Bursera simaruba*; Area de Conservacion Guanacaste, Potrerillos, Rio Azufrado, 95 m, 29 Sep. 2002 (1 female), G. Pereira, host: *Bursera simaruba*; Area de Conservacion Guanacaste, Santa Rosa, Quebrada Guapote, 240 m, 12 July 1994 (1 female), 280 m, 7 July 1993 (3 males, 1 female), gusaneros, host: *Bursera tomentosa*; Area de Conservacion Guanacaste, Santa Rosa, Area Administrativa, 295 m, 4 May 1995 (5 males, 14 females), genitalia slide male USNM 136087, gusaneros, 22 Aug. 1984 (1 female), D.H. Janzen, host: *Bursera simaruba*; Area de Conservacion Guanacaste, Santa Rosa, Bosque San Emillio, 300 m, 30 June 1983 (1 male), 7 July 1983 (2 males), D.H. Janzen, host: *Bursera tomentosa*; Area de Conservacion Guanacaste, Santa Rosa, Laguna Escondida 285 m, 23 June 2005 (1 male, 1 female), R. Franco, host: *Bursera tomentosa*; Area de Conservacion Guanacaste, Santa Rosa, Bosque Humedo, 290 m, 21 Aug. 1991 (1 male), gusaneros, host: *Bursera tomentosa*; Area de Conservacion Guanacaste, Santa Rosa, Luces, 6 July 1992 (1 female), gusaneros, host: *Bursera tomentosa*; Area de Conservacion Guanacaste, Pocosol, Casa Garzal, 245 m, 1 July 2004 (1 male), R. Franco, host: *Bursera simaruba*; Area de Conservacion Guanacaste, Cacao, Sendero Guayabal, 500 m, 7 Oct. 2004 (1 male, 1 female), D. Garcia, host: *Bursera simaruba*. **CUBA: **LA HABANA: Santiago de Las Vegas, 12 July 1931 (1 female), genitalia slide USNM 136068, A. Otero. ORIENTE: Santiago, (1 female), genitalia slide USNM 33943. **DOMINICAN REPUBLIC:** BARAHONA: nr. Filipinas, Larimar Mine, 20–26 June 1997 (5 males, 1 female), genitalia slide male MGP 1334, P. Landolt, R. Woodruff, P. Skelley [LAN]. DAJABON:13 km S Loma de Cabrera, 400 m, 20–22 May 1973 (2 females), D. & M. Davis. LA VEGA: Hotel Montana, 520 m, 28 May 1973 (2 males, 1 female), genitalia slide male USNM 135936, D. & M. Davis; Constanza, Hotel Nueva Suiza, 1164 m, 29 May 1973 (1 female), D. & M. Davis; vic. Jarabocoa, 22 June 1981 (1male), 27 June 1981 (1 male), genitalia slide USNM 136266, C.V. Covell, Jr. NATIONAL DISTRICT: Santo Domingo, (1 female), A. Busck. **ECUADOR:** GALAPAGOS: Indefatigable, Conway Bay, 1 Apr. 1923 (1 male, 3 females), genitalia slide male USNM 135966; South Seymour, 23 Apr. 1923 (1 male). IMBABURA: Paramba, Jan. –May (1 male), genitalia slide MGP 1309 [BMNH]. **GUATEMALA:** BAJA VERAPAZ: Chejel, Schaus and Barnes Coll. (1 male), genitalia slide, USNM 135915. SUCHITEPEQUEZ: Univ. del Valle de Guatemala Research Station, nr. Aldea Adelaida/Finca Panama, nr. Santa Barbara, 1550 m, 12 Aug. 2010 (1 male), P.J. Landolt [LAN]. ZACAPA: Santa Cruz, Marble Quarry rd., NE of Teculutan, 560 m, 18 July 2007 (3 males, 1 female), genitalia slide MGP 1339, 290 m, 19 July 2007 (2 males), genitalia slide MGP 1342, P.J. Landolt [LAN], (1 male), R.S. Zack [WSU]. **MEXICO:** DISTRICTO FEDERAL: Mexico City, (1 male), C. Mayer [BMNH]. HIDALGO: 5 mi E Tulancingo, 7400 ft., 24 July 1963 (1 male), genitalia slide USNM 136055, Duckworth & Davis; nr. San Vicente, 2 July 1965 (4 males), genitalia slide USNM 135954, Flint & Ortiz; Zacualpan, 15 Aug. (1 male, 2 females), genitalia slide male USNM 33942, R. Muller. JALISCO: Guadalajara, Coll. Wm. Schaus (1 female); Guadalajara, Oct. –Nov. 1898 (1 male), P. H. Goldsmith, Oct. 1896 (1 male), Schaus [BMNH]. OAXACA: Oaxaca, (1 male, 1 female), genitalia slide male USNM 42805, Coll. Wm. Schaus, June 1896 (1 male), Schaus [BMNH]. PUEBLA: Tehuacan, 11 June (1 female), R. Muller. TAMAULIPAS: Rancho del Cielo, 6 km NNW Gomez Farias, 3500 ft., July 1982 (1 female), genitalia slide USNM 135056, M.A. Solis. VERACRUZ: Orizaba, 11 June (1 male), R. Muller; Jalapa, (1 male), genitalia slide MGP 1326, M. Trujillo [BMNH]. YUCUTAN: Chichen Itza, 7 July 1955 (1 female), E. C. Welling [BMNH]. **U.S.A.:** FLORIDA: Collier Co., Chokoloskee, (1 male, 1 female), genitalia slides m USNM 136256, f USNM 136262. Hernando Co.: Bay Port, 24 Jan. 1989 (7 males, 2 females), genitalia slides male MGP 1274, 1277, 1281, J. Gillmore MGCL. Lee Co.: no specific locality, 18 Sep. 1987 (1 male), genitalia slide USNM 136052, D. Maloney USNM. Levy Co.: Cedar Key, 20 Sep. 1995 (1 male, 2 females), genitalia slide m MGP 1280, J. Gillmore & J. Medal MGCL. Manatee Co., Oneco, May 1954 (1 female), genitalia slide USNM 136258, P. Dillman. Miami-Dade Co.: Royal Palm State Park, (1 male, 1 female), Mar. (1 female), genitalia slides female USNM 136041, 136261, F.M. Jones; Oweissa-Bauer Hammock, 27 Dec. 1979 (1 female), genitalia slide MGP 1287, H.D. Baggett MGCL. Monroe Co.: Big Pine Key, Cactus Hammock, 20 Sep. 1989 (2 males), genitalia slides male MGP 1282, 1283, D. Habeck, J. Gillmore, M. Hennessey MGCL; Crawl Key, 22 Mar. 1988 (1 male), genitalia Vial #83, D.H. Habeck MGCL; Fleming Key, 20 June 1979 (1 male, 1 female), J.A. Acree & H.V. Weems, Jr. MGCL; Key Largo, 16 Sep. 1964 (1 female), Mrs. Spencer Kemp MGCL; Key Largo Key [sic], 20 Sep. 1964 (1 male, 1 female), genitalia slide male MGP 1278, Mrs. Spencer Kemp MGCL; Long Key State Park, 21 Dec. 1983 (1 male), T.S. Dickel TDC; No Name Key, 29 July 1992 (1 male), W.L. Adair, Jr. MGCL. Pinellas Co.: Dunedin, Hammock Park, 19 Jan. 1986 (1 female), 2 Feb. 1986 (1 female), 8 Feb. 1986 (1 female), J.D. Worsley MGCL. Sarasota Co.: Siesta Key, 3 Jan. 1960 (1 male), 21 Nov. 1953 (1 female), genitalia slide USNM 136259, C.P. Kimball USNM, 2 Apr. 1954 (1 female), genitalia slide MGP 1286, 18 May 1957 (1 female), 18 May 1960 (1 female), 5 Nov. 1953 (1 female), C.P. Kimball MGCL; St. Lucie Co.: 8 mi N Ft. Pierce n Turnpike, 22 Sep. 1995 (1 male), D.H. Habeck, R. Goodson, G. McDermott MGCL. **VENEZUELA:** ARAGUA: Rancho Grande, 1100 m, 30–31 Mar. 1978 (1 male, 1 female), 1–3 Apr. 1978 (3 males, 1 female), genitalia slide male USNM 135964, J.B. Heppner, 22–31 July 1967 (7 males, 5 females), genitalia slide male USNM 135963, genitalia slide female USNM 135960, 1–7 Aug. 1967 (7 males, 3 females), 8–14 Aug. 1967 (9 males, 5 females), 15–21 Aug. 1967 (1 male, 2 females), genitalia slide male USNM 42804, R.W. Poole. LARA: Yacambu Nat. Park, 13 km SE Sanare, 1560 m, 28–31 July 1981 (2 males), 1–5 Aug. 1981 (3 males), Genitalia slide USNM 135968, J. Heppner. MERIDA: Mucy Fish Hatchery, 7 km E Tabay, 6600 ft., 10–13 Feb. 1978 (1 male), J.B. Heppner. NORTE DE SANTANDER: Cucuta, (1 male), genitalia slide MGP 1305 [BMNH]. T.F. AMAZONIA: Cerro de la Neblina, Basecamp, 140 m, 1–10 Mar. 1984 (1 female), D. Davis & T. McCabe. YARACUY: Aroa, (1 male, 1 female), Coll. Wm. Schaus.

#### Diagnosis.

**Figures 9–16.**
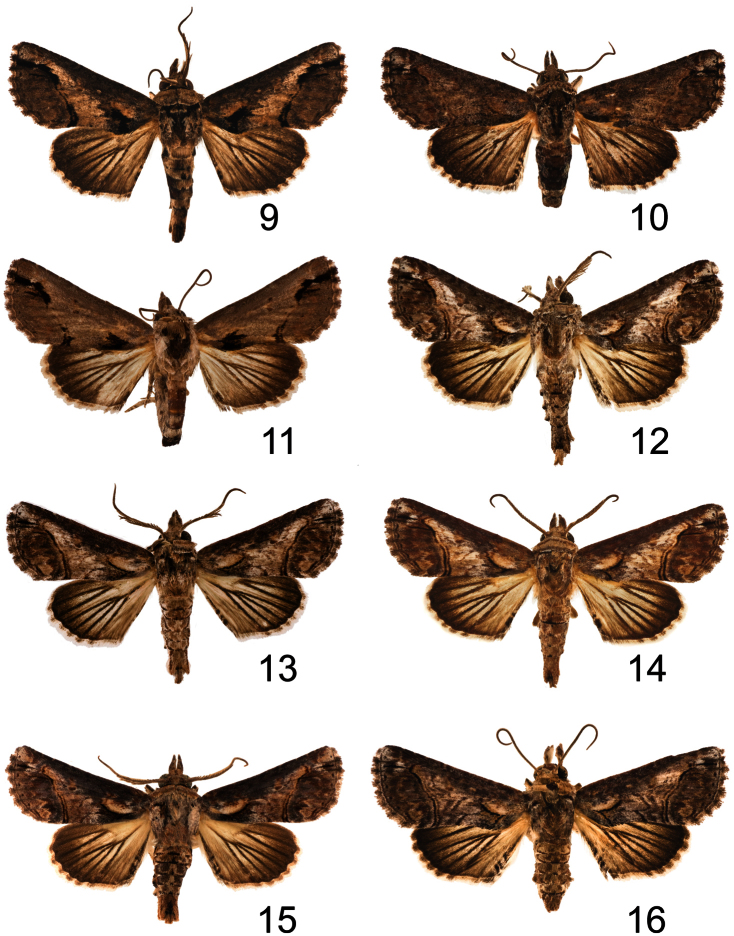
*Paectes* adults. **9** *Paectes nana* ♂, nr. San Vicente, Hidalgo, Mexico, 2 July 1965, Flint & Ortiz **10** *Paectes nana* ♀, Rancho Grande, Aragua, Venezuela, 1100 m, 8–14 Aug. 1967, R. W. Poole **11** *Paectes nana* ♀, Rancho Grande, Aragua, Venezuela, 1100 m, 8–14 Aug. 1967, R. W. Poole **12** *Paectes asper* ♂, Grand Savane, Dominica, 31 Oct. 1966, E. L. Todd **13** *Paectes asper* ♂, Santiago, Cuba **14** *Paectes asper* ♂, Santiago, Cuba **15** *Paectes asper* ♂, Palm Beach, Florida, Dec. 1898, R. Thaxter **16** *Paectes asper* ♀, Nassau, New Providence, Bahamas, J. Doll

**Figures 17–24.**
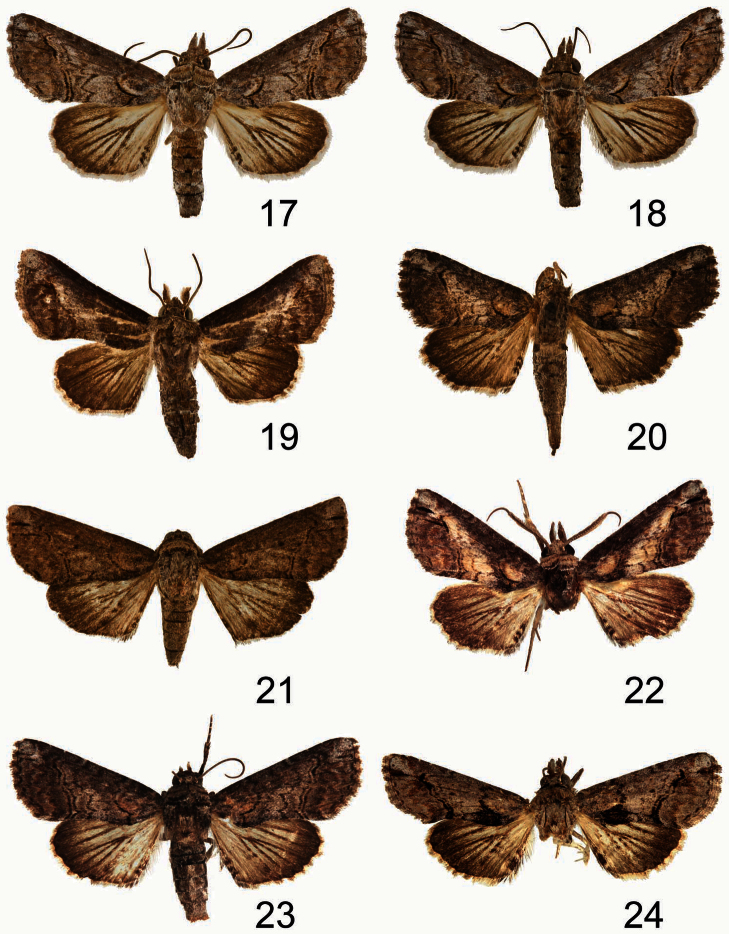
*Paectes* adults. **17** *Paectes asper* ♀, Grand Savane, Dominica, 14 June 1964, O. S. Flint, Jr. **18** *Paectes asper* ♀, 1 mi N Mahaut, Dominica, 12 June 1964, O. S. Flint, Jr. **19** *Paectes asper* ♀, Haiti **20** *Paectes medialba* ♂, Holotype, Tucuman, Argentina, R. Schreiter **21** *Paectes medialba* ♀, Tucuman, Argentina, Mar. 1905, E. Dinelli **22** *Paectes sinuosa* ♂, Salta, Argentina, Feb. [19]05, J. Steinbach **23** *Paectes sinuosa* ♂, Suncho Corral, Santiago del Estero, Argentina, J. Steinbach **24** *Paectes sinuosa* ♀, Sara, Santa Cruz, Bolivia, 450 m, Jan., J. Steinbach.

*Paectes nana* has two distinct forms. The most easily recognized bears exaggerated dark markings on the apical portion of the postmedial line that is contiguous with the subapical dash, the posterior portion of the postmedial line from CuA1 to posterior margin, and the antemedial line from just dorsal to anal vein to posterior margin (Fig. 11). This form is not present in *Paectes asper*. The other form of *Paectes nana* most resembles both *Paectes asper* (Figs 8–10). The forewing costa in *Paectes nana* is gray with small, faint, dark-gray quadrate spots along the margin at approximately 1/4 and 1/2 length of wing. In *Paectes asper* the forewing costa is ferruginous mixed with some gray and the quadrate spots are absent (Figs 12–15). In females of *Paectes nana* (Figs 10–11), the medial area of the forewing is gray, or can have gray scales tipped with white, giving a slightly lighter overall color than the remainder of the forewing. In *Paectes asper*, the medial area is somewhat lighter in coloration than the remainder of forewing and entirely white scales are present as are white-tipped gray scales (Figs 16–18). A cream-colored basal spot is present and contrasts with the remainder of the forewing in *Paectes asper*, and in *Paectes nana* the basal area is only very slightly contrasting with white-tipped gray scales.

Both the male and female genitalia are distinct between these species. In the male genitalia, the costa is thumb-like with a more produced apex in *Paectes nana* (Fig. 32), whereas in *Paectes asper* the costa in truncate and the apex is not produced (Fig. 33). In *Paectes nana*, there are several flat setae arising from a sclerotized ridge on the dorsal surface of the valve (Fig. 32), these flat setae are absent in *Paectes asper* (Fig. 33). The cornutus at the base of the vesica is wide in *Paectes nana* (Fig. 32), but narrow in *Paectes asper* (Fig. 33). In the female genitalia, *Paectes nana* is easily recognized by the ostium bursae being a sclerotized circle (Fig. 39), but in *Paectes asper* the ostium is crescent shaped (Fig. 40).

#### Redescription.

**Male.** Sexes dimorphic. *Head* – vertex with broad scales, mixture of cream-colored and light-brown scales, anterior margin with a few black scales; labial palp porrect, a mixture of gray, light-brown, dark-ferruginous, and black scales, internal surface white; eyes large, globular; frons with broad scales, projecting slightly beyond anterior eye margin, with cream-colored and light-brown scales with a few black scales medially; male antenna broadly bipectinate to 2/3 length then filiform. *Thorax* – prothorax concolorous with vertex, anterior margin with a thin black line; patagium concolorous with prothorax, mixed with hairlike scales; protibia white mixed with black, apical band white, obscure, tarsi black with distinct white apical bands; middle tibia grayish brown, tarsi gray with white apical bands; hind tibia cream-colored, tarsi cream colored; underside with white hairlike scales; forewing length 10.9–11.6 mm; costal area a mixture of dark-gray scales tipped a lighter color and a few black scales; distinct ovate cream-colored basal spot margined posteriorly with a few black scales; thin black antemedial line from posterior margin to middle of basal spot; interior of wing from distal margin of ovate spot to postmedial line mostly white and contrasted with subterminal and terminal areas; reniform spot obscure, with only a few pale-ferruginous scales; postmedial line black, a double line from posterior margin to vein M2 then single until merging with black dash between veins R5 and M1 that extends to outer margin; apical spot white; subterminal area brown, veins gray, color extending on to fringe; terminal line a series of black, shallow scalloped lines between veins; fringe brown, gray patches from wing veins resulting in a somewhat checkered appearance; hind wing white, marginal shading dark gray, veins highlighted dark gray, anal fold a white and dark gray striped pattern. *Abdomen* – cream colored scattered with a few pale-ferruginous scales; male eighth segment membranous with a pair of short, sternal, sclerotized bars and a pair of longer, wider, dorsal sclerotized bars; a pair of lateral coremata bearing numerous, fine, elongate setae. *Genitalia* (Fig. 32) – Uncus triangulate, apex recurved and pointed; subscaphium longer than uncus, triangulate, decurved, apex pointed; valve membranous, elongate, narrowed distally, apex round, covered with many elongate setae, basal-dorsal margin sclerotized, with several wide, spine-like setae; costa of valve short, deeply curved, apex produced and rounded, densely covered with elongate setae; sacculus well developed, proximal half fused with valve, distal half free, elongate, curved inward, longer than valve, apex round; saccus triangulate; aedeagus straight, slightly bent at distal third, dorsum in distal third covered with minute spicules; base of vesica a short tube with one flat, elongate cornutus with pointed apex directed posteriorly, vesica ovate, small round diverticulum just distal to flat basal cornutus, apex of vesica with an irregular sclerotized area bearing a short, thumb-like cornutus. **Female.** As in male except: *Head* –antenna filiform; forewing length 9.4–9.9 mm; ground color pale gray; antemedial line black, reduced to a concave line from just below Cu vein to anal vein connected to a convex line from anal vein to posterior margin; basal spot absent; interior of wing from base to postmedial line pale gray with scattered white scales or scales tipped white and only slightly paler than subterminal and terminal areas; medial line black, faint, dentate from just below Cu vein to posterior margin. *Genitalia* (Fig. 39) – Papillae anales truncate, soft, fleshy, covered with numerous setae; ninth sternite covered with minute spicules distally with spicules becoming larger and thicker closer to ostium bursae; anterior apophyses fused with eighth segment; posterior apophyses present; ostium bursae with sclerotized, crescent-shaped large dorsal and small ventral caps; base of ductus bursae, as it emerges from ostium bursae, sclerotized then becomes membranous and striated, after splitting with appendix bursae, ductus bursae narrower and more heavily striated; appendix bursae ovate, membranous; corpus bursae ovate, covered internally with numerous thornlike signa.

#### Distribution and biology.

*Paectes nana* is widespread from Florida through the Greater Antilles, except for Puerto Rico, and from Mexico to Costa Rica; in South America distributed from Venezuela, Colombia, and northern Ecuador (Fig. 50). It has been introduced to the Galapagos Islands (Roque-Álbelo and Landry 2011).

*Paectes nana* is a native species from Florida that has been reared from Brazilian peppertree in several counties, including Hernando, Lee, Levy, Monroe, and St. Lucie. Larvae that were collected in September and October had a pupal stage from 9–18 days and larvae collected in January and February had a pupal stage from 11–15 days. Adults probably fly all year with recorded dates from January–March, June–July, September–October, and December. Dyar (1901) stated that larvae of *Paectes nana* (referred to as *Paectes burserae*) are common on gumbo-limbo (*Bursera simaruba* (L.) Sarg., Burseraceae). In Costa Rica *Paectes nana* collecting dates range from May through November and has been reared from *Bursera simaruba* and *Bursera tomentosa* (Jacq.) Triana & Planch.

#### Remarks.

*Paectes nana* has two forms. A form that is easily confused with *Paectes asper* and a more boldly marked form where the antemedial and postmedial lines and marginal dash are heavily marked with black and there are scattered black scales along the forewing posterior margin adjacent to the antemedial line. The holotype of *Paectes nana* is a heavily marked form.

### Paectes asper

Pogue sp. n.

urn:lsid:zoobank.org:act:16EDE70C-D0AF-4CF7-A020-27E4EB09AF8F

http://species-id.net/wiki/Paectes_asper

Figs 12 –19 33 40 51

#### Type material. 

**Holotype** male – **CUBA**: Santiago, Collection Wm. Schaus; HOLOTYPE / *Paectes asper* Pogue” [red label]. USNM. Paratypes – (134 males, 85 females). All from USNM unless noted. Same data as holotype (9 males, 9 females) genitalia slide male USNM 135978, genitalia slides female USNM 135977, 135981– 135983; (2 males), genitalia slide male MGP 1314 [BMNH]. **BAHAMAS**: no specific locality (1 male, 1 female) [BMNH]. ABACO ISLANDS: no specific locality, (2 males, 2 females), Mar. 1902 (1 male), genitalia slide male MGP 1313, J.J. Bonhote [BMNH]. ANDROS: Andros Town, 27–29 Jan. 1965 (1 male), genitalia slide USNM 135927, leg. W. U. R. Piath; Mangrove Cay, 11 Jan. 1902 (1 female), J.J. Bonhote [BMNH]. NEW PROVIDENCE: Nassau, (1 female), Col. Jacob Doll.; Nassau I., 8 July 1898 (2 males, 1 female), 14 July 1898 (3 females), J.J. Bonhote [BMNH]. **BRITISH VIRGIN ISLANDS**: Great Camanoe Is., 1/3 mi ESE Cam Bay, 18 Mar. 1974 (1 male), C.L. Remington; Guana Island, North Bay, 0 m, 15–25 July 1986 (1 female), S.E. Miller & M.G. Pogue; Guana Island, 0-80 m, 13–26 July 1986 (1 male), genitalia slide USNM 135931, S.E. Miller & M.G. Pogue; Guana Island, 1–14 July 1984 (11 males, 8 females), Genitalia slides male USNM 135979, 135990, 135992, 136009, genitalia slides female 135998, 136005, 136006, 136007, 9–15 July 1985 (1 female), S.E. and P.M. Miller; Tortola, 14 May 1980 (1 female), 29 May 1980 (1 female), 28 July 1973 (1 female), 23 Oct. 1972 (1 female), Oct. 1972 (3 males), genitalia slides MGP 1319, 1320, 12 Nov. 1973 (1 male), 14 Nov. 1972, (1 female), 18 Nov. 1972 (1 female), J. Lorimer, 5 June 1974 (1 female) [BMNH]. **CAYMAN ISLANDS:** CAYMAN BRAC: behind Stakes Bay, 20 May 1938 (3 females), 21 May 1938 (1 female), 22 May 1938 (1 male), C.B. Lewis, G.H Thompson; N. coast of Stakes Bay, 20 May 1938 (1 male, 1 female), 22 May 1938 (1 male), genitalia slide MGP 1318, C.B. Lewis, G.H Thompson; west end of Cotton-tree Land., 19 May 1938 (1 male), 22 May 1938 (1 male), C.B. Lewis, G.H Thompson [BMNH]. GRAND CAYMAN: east end of East End, 13 May 1938 (1 male), 16 May 1938 (1 female), C.B. Lewis, G.H Thompson; Georgetown, (2 males, 2 females), genitalia slide male MGP 1317, A.W. Cardinall; N. coast of North Side, 11 July 1938 (1 female), 14 July 1938 (1 female), 16 July 1938 (1 female), C.B. Lewis, G.H Thompson; west end of Georgetown, 14 May 1938 (1 female), C.B. Lewis, G.H Thompson [BMNH]. LITTLE CAYMAN: south coast of South Town, 31 May 1938 (2 males), 2 June 1938 (1 male, 1 female), 4 June 1938 (1 female), C.B. Lewis, G.H Thompson [BMNH]. **COLOMBIA:** SAN ADRES, PROVIDENCIA, AND SANTA CATALINA: San Andrés, 300 ft., Apr. 1926 (2 males), genitalia slides MGP 1328, 1351, F.W. Jackson [BMNH]. **CUBA:** no specific locality, (10 males, 2 females), genitalia slide male USNM 42806, genitalia slides female USNM 135962, 135985, Coll. Wm. Schaus, (1 male), Dognin Coll.; no specific locality, (4 males, 4 females), genitalia slide male MGP 1315 [BMNH]. GUANTANAMO: Baracoa, (3 males, 1 female), Aug. Busck Collector, 12 Feb. 1958 (1 male), Genitalia slide USNM 135955, B. Wright. HOLGUIN: Holguin, (2 males, 2 females), H.S. Parrish [BMNH]. LA HABANA: Cayamas, (1 male), E.A. Schwarz. ORIENTE: Santiago, (1 male, 1 female), genitalia slide male MGP 1314, W. Schaus [BMNH], June 1902 (1 male), Nov. 1902 (1 male), W. Schaus [BMNH]. **DOMINICA:** 1 mi N Mahaut, 12 June 1964 (1 female), genitalia slide USNM 136002, O.S. Flint, Jr.; Clarke Hall, 3 June 1964 (1 female), genitalia slide USNM 135984, O.S. Flint, Jr.; Grande Savane, 13 May 1964 (1 female), genitalia slide USNM 135961, 20 May 1964 (1 male, 1 female), genitalia slide male USNM 135975, genitalia slide female USNM 136057, 14 June 1964 (1 female), genitalia slide USNM 135995, 31 Oct. 1966 (1 male, 1 female), genitalia slide male USNM 135994, genitalia slide female USNM 136008, O.S. Flint, Jr.; Macoucheri, 1 Feb. 1965 (1 male), genitalia slide USNM 136058, 12 Feb. 1965 (1 male, 1 female), genitalia slide female USNM 42810, 5 Mar. 1965 (1 male), J.F.G. & Thelma Clarke. **DOMINICAN REPUBLIC:** San Cristobal, 8–9 June 1969 (1 male), genitalia slide USNM 135986, Flint & Gomez. **HAITI**: No specific locality, (2 males, 1 female), genitalia slide male USNM 135928; no specific locality, (2 males, 1 female), genitalia slide male MGP 1322 [BMNH]. **JAMAICA**: no specific locality, (3 males), genitalia slide male USNM 135930; no specific locality, (6 males, 6 females) [BMNH]. ST. ANDREW: Newcastle, (1 male), genitalia slide MGP 1316 [BMNH]. ST. JAMES: Montego Bay, 24 Jan. 1924 (1 male, 1 female), Gillett; Up Camp (1 male) [BMNH]; Kingston, July 17, at electric light, several were taken, Cockerell (1 male). TRELAWNY: Runaway Bay, 28 Mar. 1905 (1 male) [BMNH]. **PUERTO RICO:** no specific locality, (1 male), genitalia slide MGP 1331 [BMNH]. **U.S.A.:** FLORIDA: Miami-Dade Co., Biscayne Bay, (1 male), Collection H.G. Dyar; Coconut Grove, Nov. 1897 (1 male), Roland Thaxter Coll. Florida City, 9 June 1937 (1 female); Miami, (5 males, 1 female), genitalia slide male USNM 136000, genitalia slide female USNM 136001. Monroe Co., Key Largo Key [sic], 13 Dec. 1968 (1 male), genitalia slide MGP 1285, Mrs. Spencer Kemp MGCL, 6 Jan. 1969 (1 female), genitalia slide USNM 136260, Mrs. Spencer Kemp USNM; Bahia Honda State Park, 6 Jan. 1989 (1 male), 17 Jan. 1990 (1 male), 21 Jan. 1996 (1 male, 1 female), 12 Mar. 1989 (1 male), 23 Mar. 1990 (1 male), 29 Mar. 1990 (1 male), 28 Oct. 1988 (1 male), 8 Nov. 1988 (1 male), 29 Dec. 1989 (1 male, 1 female), T.S. Dickel TDC; Long Key State Park, 5 Feb. 1986 (1 male), 16 Feb. 1985 (1 male), 4 Mar. 1994 (1 male), 26 Dec. 1994 (1 male), T.S. Dickel TDC; Key Largo Hammock Botanical State Park, 17 Jan. 1987 (1 male), 30 Jan. 1992 (1 male), 2 Feb. 1995 (1 male), 12 Feb. 1990 (1 male), 21 Feb. 1995 (2 males), T.S. Dickel TDC; No Name Key, 19 Oct. 1987 (1 male), T.S. Dickel TDC; Windley Key, 3 June 1983 (1 male), T.S. Dickel TDC. Palm Beach Co., Dec. 1897 (1 male), genitalia slide USNM 135932, Dec. 1898 (1 male), genitalia slide USNM 135999, R. Thaxter. Palm Beach Co., Palm Beach, Dec. 1897 (1 female), R. Thaxter. **U.S. VIRGIN ISLANDS:** ST. CROIX: Blue Mtn., 6–16 July (1 male), E.L. Todd; Christiansted, 19 Nov. 1941 (1 male, 1 female), H.A. Beatty; Gallows Point, 11 July 1956 (1 male), genitalia slide USNM 136044, J.G. Coutsis; Kingshill, 6–16 July 1967 (1 male), E.L. Todd; Mt. Eagle, 6–16 July 1967 (1 female), E.L. Todd; Orangegrove, W End, 6–16 July 1967 (3 males, 2 females), Genitalia slide m USNM 42807, E.L. Todd. USNM, CNC, BMNH

#### Etymology.

The species name is the Latin term for rough, which refers to the roughened texture of the apex of the free saccular extension in the male genitalia.

#### Diagnosis. 

The forewing costa is ferruginous with some gray in *Paectes asper* and mostly gray with some ferruginous in *Paectes arcigera*. The medial area of the forewing is mostly white with a white apical spot in *Paectes asper*; in *Paectes arcigera* these areas are cream-colored.

The male genitalia are easily differentiated between *Paectes asper* and *Paectes arcigera*. The valve in *Paectes asper* has thick, curved dorsal setae; in *Paectes arcigera* the setae are hairlike. The costa is truncate in *Paectes asper* but triangulate in *Paectes arcigera*. The free saccular extension is narrow in *Paectes asper* and wide with an expanded apex in *Paectes arcigera*.

The female genitalia have small, curved lateral projections at the base of the eighth sternite in *Paectes asper*; these projections are absent in *Paectes arcigera*. The ostium bursae is crescent shaped bearing minute spicules in *Paectes asper* whereas in *Paectes arcigera* the ostium bursae is semicircular in shape and bears large conical spines.

#### Description.

**Male.** *Head* – antenna broadly bipectinate to 3/5 length then filiform; eyes large, globular; vertex with broad scales, light-brown mixed with pale- and dark-ferruginous scales; frons with broad scales, projecting slightly beyond anterior eye margin, concolorous with vertex, two small black dots on eye margin; labial palp porrect, mixture of light-brown and ferruginous scales, internal surface white. *Thorax* – prothorax pale ferruginous, with a thin, black anterior margin, posterior margin pale gray, can be mixed with black or dark-ferruginous scales; patagium pale gray variably mixed with ferruginous and a few black scales, mixed with hairlike scales; pro and mid tibia gray and ferruginous mixed with white scales, apical band present, tarsi ferruginous with white apical bands; hind tibia ferruginous mixed with white scales, lighter than pro or mid tibia, tarsi white mixed with ferruginous scales, apical bands not distinct; underside with white hairlike scales; forewing length 9.4–12.9 mm; costal area dark gray and ferruginous; ovate basal spot distinct; antemedial line black, sharply angulate basally, continues around ventral margin of ovate spot, arrowhead shaped; reniform a pair of small ferruginous spots, vertically oriented; interior of wing a variable mix of white, pale- ferruginous, and ferruginous scales, always lighter than costa and subterminal area; postmedial line black, black and ferruginous, or ferruginous, a double line from posterior margin to below M vein, then a single line to M1 vein; black horizontal dash between R5 and M1 vein continuing to outer margin; apical spot white; subterminal area gray, distal border ferruginous and dentate; terminal area with irregularly shaped tan patch near tornus; terminal line a series of dark-ferruginous spots between wing veins; fringe pale gray becoming white at apex; hind wing white, marginal shading dark gray, veins highlighted dark gray, anal fold with a white and dark-gray striped pattern; fringe white. *Abdomen* – dorsum variable from pale gray to dark gray mixed with ferruginous scale patches, distal margin of segments usually with a darker line that can be ferruginous or black, obscure cream-colored dorsal band from middle to antemedial segment; venter variable from white to tan to ferruginous, medial line black to ferruginous flanked by paler, wider, lateral lines variable in color and intensity from black to gray, or can be represented by a thin medial line; male eighth segment membranous with a pair of short, sternal, sclerotized bars and a pair of longer, slightly wider, dorsal sclerotized bars; a pair of lateral coremata bearing numerous, fine, elongate setae. *Genitalia* (Fig. 33) – Uncus triangulate, apex sharply recurved and pointed, 0.62–0.64 × length of subscaphium; subscaphium, triangulate, decurved, apex pointed; valve membranous, elongate, narrow, covered with broad, curved setae; costa sclerotized, deeply recurved, apex rounded or truncate, sparse hairlike setae dorsally and at apex; sacculus well developed, proximal half fused with valve, distal half free, elongate, broad, curved inward, longer than valve, apex with roughened surface; saccus V-shaped; aedeagus straight; vesica ovate, large bulbous lateral diverticula just above vesica base; adjacent to basal diverticulum a second bulbous diverticulum with an irregular sclerotized patch bearing elongate somewhat flattened cornutus; thumblike diverticulum opposite sclerotized patch; ventral cornutus at base of vesica, stout, elongate, pointed caudally. **Female.** As in male except:antenna filiform; forewing length 9.2–12.5 mm; medial area of forewing white, suffused with pale gray scales and less contrasting than in male. *Genitalia* (Fig. 40) – Papillae anales ovate, soft, fleshy, covered with numerous setae; venter of eighth segment covered with minute spicules; anterior apophyses fused with eighth sternite; posterior apophyses present; ventrolateral corners of eighth sternite produced into small, outwardly curved projections; ostium bursae crescent shaped, sclerotized plate bearing minute spicules; ventral to ostium bursae, base of ductus bursae rectangular and fused to eighth sternite, short membranous section of ductus bursae at juncture of appendix bursae and ductus bursae, ductus bursae striate and gradually enlarges into corpus bursae; appendix bursae emerges from ductus bursae just ventral to rectangular part of ductus bursae, consists of two sacs, one at junction of ductus bursae then a constriction and a second, enlarged, membranous sac; corpus bursae tear-drop shaped, covered internally with numerous thornlike signa.

#### Distribution and biology.

*Paectes asper* is distributed from southern Florida and Bahamas to the Greater Antilles (except Puerto Rico), and the British Virgin Islands, U.S. Virgin Islands, and Dominica in the Lesser Antilles (Fig. 51). A specimen in the BMNH is labeled Costa Rica, St. Andrews I. This specimen was interpreted to be from San Andrés Island in the western Caribbean, which is now part of Colombia.

#### Remarks.

*Paectes asper* was in the series of specimens in the USNM collection identified as *Paectes obrotunda*. Specimens of *Paectes asper* can be confused with specimens of *Paectes arcigera* from the British Virgin Islands, U.S. Virgin Islands, and Dominica. *Paectes asper* is sympatric with *Paectes arcigera* on Guana Island, B.V.I., St. Croix, U.S.V.I., and Dominica. In Florida, Cuba, and the Dominican Republic, *Paectes asper* can be confused with *Paectes nana*.

### Paectes medialba

Pogue sp. n.

urn:lsid:zoobank.org:act:8C58724A-B96F-42FE-B82B-E28C70FB8341

http://species-id.net/wiki/Paectes_medialba

Figs 20–21 34 41 49

#### Type material.

**Holotype** male – **ARGENTINA:** Tucuman, R. Schreiter Collr., Collection Wm. Schaus, USNM ENT 00148677, genitalia slide, USNM 136027 [green label]; HOLOTYPE / *Paectes medialba* Pogue” [red label]. USNM. Paratype 1 female – **ARGENTINA:** La Rioja, genitalia slide, USNM 136067. USNM

#### Etymology.

The species name is derived from the combination of the Latin terms *medius* (middle) and *albus* (white) to refer to the white medial area of the male forewing.

#### Diagnosis.

*Paectes medialba* has been confused with *Paectes longiformis* in the USNM collection. It differs from *Paectes longiformis* by the more pronounced white medial area of the male forewing and its distribution in northwestern Argentina versus the northeastern Brazil distribution of *Paectes longiformis*. There are many differences in the male genitalia of *Paectes medialba* and *Paectes longiformis*. The free extension of the sacculus in *Paectes longiformis* is much longer than in *Paectes medialba* and is spiculate in *Paectes medialba* and non-spiculate in *Paectes longiformis*. The setae on the dorsal surface of the valve are wide, elongate, curved apically, and numerous in *Paectes medialba* whereas in *Paectes longiformis* they are hairlike, shorter, straight, and more sparse. In the female genitalia the lateral projections of the eighth sternite are more produced and sharply pointed in *Paectes medialba* (Fig. 41) than in *Paectes longiformis* (Fig. 38). The ductus bursae between its juncture with the appendix bursae and its entering the corpus bursae is tapered in *Paectes medialba* but straight in *Paectes longiformis*. The signa are more numerous in the corpus bursae of *Paectes longiformis* than in *Paectes medialba*.

#### Description.

**Adult.** Sexes dimorphic. **Male.** *Head* – antenna broadly bipectinate to just beyond half length then filiform; eyes large, globular; vertex with broad scales, pale gray, finely tipped with white; frons with broad scales, badly rubbed, pale gray with black posterior margin; labial palp porrect, mixture of pale-gray and black scales tipped with white, internal surface white. *Thorax* – prothorax mixture of gray and white scales, anterior margin a thin black line; patagium pale gray with dark-gray scales medially, all tipped with white, mixed with hairlike scales; protibia black scales tipped with white, more white scales along inner margin, tarsi black barely tipped with white, white apical bands; middle and hind legs absent from holotype; forewing length 9.4 mm; costal area dark gray mottled with white scales, faint white dashes along costa from just beyond middle to below apex; distinct ovate basal spot, few dark gray and white scales basally, remainder of scales tan; thin black antemedial line from posterior margin to Cu vein, forming ventral border of basal spot; medial area mostly white mixed with a few tan and dark-brown scales; medial line dark gray, faint, straight from posterior margin to anal vein, curved from anal vein to Cu vein; reniform spot obscure, a pair of tiny dots, ventral dot just above Cu vein in discal cell, dorsal dot just below R vein, dark brown; postmedial line black, angulate, from posterior margin to vein R5, faint medially; black dash along vein R5 not quite extending to outer margin and not contiguous with postmedial line; white apical spot mixed with a few dark-gray scales; subterminal area brown, scattered with white scales; terminal line a series of dark-brown, recurved lines between veins; fringe brown; hind wing white, marginal shading dark gray, veins highlighted dark gray, anal fold with a white and dark-gray striped pattern. *Abdomen* – eighth segment membranous with pair of dorsal sclerotized bars; pair of lateral coremata bearing numerous, fine, elongate setae. *Genitalia* (Fig. 34) – Uncus triangulate, apex recurved and pointed, 0.7 × length of subscaphium; subscaphium triangulate, decurved, apex pointed; valve membranous, rectangulate, apex produced, round, dorsal surface covered with broad, apically curved, elongate setae; costa thumb shaped, dorsal margin curved, elongate setae dorsally in distal third; sacculus well developed, proximal half fused with valve, distal half free, angulate, spiculate, shorter than valve, apex curved inward; saccus V-shaped, arms wide; aedeagus straight, dorsally spiculate in apical 2/3; vesica uninflated, a single, basal leaf-like cornutus, grooved sclerotized area bearing short cornutus. **Female**. As in male except: *Head* –antenna filiform; eyes large, globular; vertex light brown, few scattered dark-brown scales; frons light brown, scattered with more dark-brown scales than vertex; labial palp porrect, brown with white-tipped scales, white internally. *Thorax* – prothorax light brown, anterior margin a thin black line, few dark-brown scales along posterior margin; patagium gray, few dark-gray scales, mixed with hairlike scales; protibia dark gray with white-tipped scales, tarsi dark gray, apical bands white; forewing length 9.9 mm; ground color brownish gray; basal area not differentiated from ground color; antemedial line black, straight from posterior margin, curved around basal area to Cu vein; medial area not differentiated from ground color; reniform spot a pair of tiny black dots on either side of Cu vein; postmedial line black, faint, double to Cu2 vein, single from Cu2 vein to R5 vein; black dash at R5 vein not contiguous with postmedial line, not quite continuing to outer margin; apical spot faint, white; subterminal area not differentiated; terminal line a black dash at tornus, then a series of black spots between veins; fringe brownish gray. *Genitalia* (Fig. 41) – Papillae anales crescent shaped, soft, fleshy, covered with numerous setae; anterior apophyses fused with eighth sternite; posterior apophyses present; venter of eighth segment spiculate in distal half, with sharply-pointed lateral projections; sclerotized ventral projection of eighth sternite enters membranous base of ductus bursae, which then becomes constricted, then widens for a short distance below constriction where appendix bursae branches, below junction of appendix bursae, ductus bursae widens as it enters corpus bursae; appendix bursae ovate, membranous; corpus bursae ovate, covered internally with numerous thornlike signa pointing inward, signa continue posteriorly into posterior part of ductus bursae.

#### Distribution and biology.

Known from northwestern Argentina (Fig. 49). Nothing is known about the biology.

#### Remarks.

*Paectes medialba* is another species that was confused as *Paectes obrotunda* in the USNM collection. It is being described to resolve confusion in this group.

### Paectes sinuosa

Pogue sp. n.

urn:lsid:zoobank.org:act:EEAB4B58-5284-418D-B865-D01A8A1CD124

http://species-id.net/wiki/Paectes_sinuosa

**Figures 25–28.**
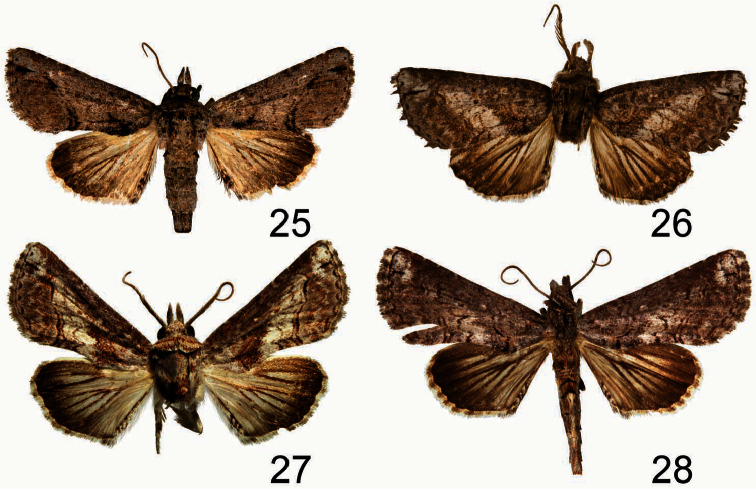
*Paectes* adults. **25** *Paectes sinuosa* ♀, Buenavista, Santa Cruz, 750 m, Bolivia, Aug.-Apr. 1906–1907, Steinbach **26** *Paectes tumida* ♂, Holotype, Geldersland, Suriname **27** *Paectes tumida* ♂, St. Jean du Maroni, French Guiana, Le Moult **28** *Paectes tumida* ♀, Bartica, British Guiana [Guyana], June 1901.

Figs 22 –25 35 42 52

#### Type material.

**Holotype** male – **ARGENTINA:** BUENOS AIRES: Los Vasquez, Dognin Collection, genitalia slide male, USNM 135916 [green label]; HOLOTYPE / *Paectes sinuosa* Pogue” [red label]. USNM. Paratypes – (22 males, 19 females) **ARGENTINA:** COLON: Sierras de Cordoba, La Granja, (1 female), A. Garcia [BMNH]. LA RIOJA: La Rioja, (1 male), genitalia slide MGP 1353, Jan.–Feb. (1 female), [no date] (1 male) F. Giacomelli [BMNH]. SALTA: Salta, Feb. 1905 (1 male), genitalia slide MGP 1312, J. Steinbach [BMNH]. SANTIAGO DEL ESTERO: Santiago del Estero, (1 female), J. Steinbach [BMNH]. TUCUMAN: Tucuman, (2 males, 1 female), genitalia slides male, MGP 1311, MGP 1350, Schreiter, 450 m, Mar. 1902 (1 female), genitalia slide MGP 1353 [BMNH]. **BOLIVIA:** SANTA CRUZ: Ichilo, Buenavista, 750 m, Aug.-Apr. 1906-1907 (1 female), Steinbach [BMNH]; Sara, 450 m, Jan. (1 female), genitalia slide MGP 1349, Nov. (1 male, 1 female), J. Steinbach [BMNH]. **BRAZIL:** AMAZONAS: Humaitá, July-Sep. 1906 (1 female), W. Hoffmanns [BMNH]. BAHIA: S. Antonio de Barra, (10 males, 1 female), [BMNH]. GOIAS: Chapada dos Veadeiros, 18–24 km N of Alto Paraiso, 1400–1500 m, 2–5 Oct. 1985 (1 male), S.E. Miller, genitalia slide USNM 135970, USNM. MINAS GERAIS: Tijuco, Dec. (1 male) [BMNH]. PARANÁ: Curitiba, 23 Apr. 1988 (1 male), L. Crestana, Genitalia slide Vial #79, MGCL; Entre Rios, (1 female) [BMNH]. PERNAMBUCCO: Serra de Communaty, Dec. 1893 (1 female), E. Gounene [BMNH]. RIO DE JANIERO: Petropolis, 1888 (1 female), Germain [BMNH]. SÃO PAULO: Alambari, 3 Sep. 1988 (1 female), 8 Dec. 1988 (1 male), 16 Dec. 1988 (1 male), L. Crestana, Genitalia slide f Vial #86, genitalia slides male MGP 1279, MGP 1288, MGCL; Porto Feliz, 1 June 1988 (1 female), L. Crestana, genitalia slide MGP 1289, MGCL. **PARAGUAY:** BOQUERON [NUEVA ASUNCION]: Nueva Asuncion, 313 m, 23–25 Mar. 1986 (1 female), M. Pogue and M. Solis, genitalia slide USNM 136014. GUAIRA: Villarrica, Dec. 1922 (1 female), J. Schade [BMNH]. PARAGUARI: Sapucaí, 24 June 1902 (1 male, 1 female), genitalia slide male MGP 1310, W. Foster [BMNH]. PRESIDENTE HAYES: Primavera, 14 Apr. 1960 (1 female), E.J. Phillips [BMNH].

#### Etymology.

The species name is derived from the Latin *sinuo* (bend), referring to the sinuate free saccular extension of the male genitalia.

#### Diagnosis.

The forewing is longer in *Paectes sinuosa* than in *Paectes medialba*. The postmedial line is double in *Paectes sinuosa* (Figs 22–25) and single in *Paectes medialba* (Figs 20–21). In the male genitalia the dorsal margin of the costa is slightly concave in *Paectes sinuosa* (Fig. 35) but greatly convex in *Paectes medialba* (Fig. 34). The free saccular extension is sinuate in *Paectes sinuosa* (Fig. 35) but straight in *Paectes medialba* (Fig. 34). At the base of the vesica there are two wide cornuti in *Paectes sinuosa* (Fig. 35) but only one in *Paectes medialba* (Fig. 34).

#### Description.

**Adult. Male.** *Head* – antenna broadly bipectinate to just beyond half length then filiform; eyes large, globular; vertex with broad scales, dark brown anteriorly, light brown posteriorly; frons with broad scales projecting slightly beyond anterior eye margin, mostly light brown with a posterior band of dark brown scales; labial palp porrect with mixture of light-brown and brown scales tipped with white, internal surface white. *Thorax* – prothorax light brown, anterior margin with thin black line; few scales forming a faint medial line; posterior margin with some dark-brown scales; patagium with dark-brown scales tipped with white, mixed with a few white scales, scales broad mixed with hairlike scales; protibia dark-brown scales tipped with white, mixed with white scales, apical band white, obscure, tarsi dark brown with distinct white apical bands; midtibia concolorous with protibia, but with more white scales giving a slightly lighter overall color, white apical band more distinct than in protibia, tarsi dark brown with white apical bands; hind tibia mostly white with a few dark-brown scales, tarsi white; underside with white hairlike scales; forewing length 10.5 mm; costal area dark gray with a few white scales forming short dashes along costa, especially from postmedial band to just below apex; distinct ovate cream-colored basal spot; thin black antemedial line from posterior margin forming ventral border to basal spot; medial area of wing white and light brown; medial line obscure, dark brown, from posterior margin to anal vein where it becomes arrowshaped then continues vertically to Cu vein; reniform spot obscure, a pair of tiny dots, ventral dot just above M vein in discal cell dark brown and larger than dorsal dot, which consists of only a few ferruginous scales; postmedial line black, double from posterior margin to vein M1; black dash between veins R5 and M1 extending to outer margin; white apical spot; subterminal area a mixture of light- brown, gray, white, and ferruginous scales, lighter than costa and darker than medial area; terminal line a series of gray, recurved lines between veins; fringe brown, gray patches opposite vein apices; hind wing white, marginal shading dark gray, veins highlighted dark gray, anal fold with a white and dark gray striped pattern. *Abdomen* – dorsum mixture of pale gray, black, and ferruginous, with small patches of black scales at caudal apex of segments 2–3; ventrum white with a faint black medial line and partial lateral lines; male eighth segment membranous with a pair of short, sternal, sclerotized bars and a pair of longer, wider, dorsal sclerotized bars; a pair of lateral, coremata bearing numerous, fine, elongate setae. *Genitalia* (Fig. 35) – Uncus triangulate, apex recurved and pointed, approximately same length as subscaphium; subscaphium triangulate, decurved, apex pointed; valve membranous, rectangulate, slightly produced distally, apex round, covered with wide, elongate setae; costa short, recurved, apex produced, round, sparsely covered with elongate, hairlike setae; sacculus well developed, proximal 2/3 fused with valve, distal 1/3 free, sinuate, spiculate, longer than valve, apex round; saccus with broad arms; aedeagus straight; vesica emerges at a right angle ventrally from aedeagus; bulbous with a short, distal diverticulum and two elongate leaflike cornuti at base, longest laterally, shortest ventrally; rectangular, grooved sclerotized plate lateral to distal diverticulum, short slightly curved cornutus on grooved plate. **Female.** As in male except:antenna filiform; forewing length 9.5 mm; antemedial line from costa to R vein dark gray, angulate toward apex then angulate toward base with a pointed apex, black from R vein to anal vein, curved with a straight line extending to posterior margin; medial area less contrasted than male with white-tipped gray scales; postmedial line black, well developed from posterior margin to CuA2 vein then faint to M2 becoming more developed from M2 to R5; thin black line along R5 to outer margin; apical spot white. *Genitalia* (Fig. 42) – Papillae anales ovate, soft, fleshy, covered with numerous setae; venter of eighth segment covered with minute spicules; anterior apophyses fused with eighth sternite; posterior apophyses present, ventrolateral corners produced into triangular projections; ostium bursae a small dimple or absent; base of ductus bursae rectangular fused to eighth sternite, ductus bursae elongate, striate, approximately same width throughout, small ventrally produced pocket just below fused base; appendix bursae emerges dorsally from approximately middle of ductus bursae, ovate; corpus bursae ovate, covered internally with numerous thornlike signa.

#### Distribution and biology.

Known from the states of Goias and São Paulo, Brazil, the Chaco of northwestern Paraguay, and in the state of Tucuman, Argentina (Fig. 52).

Specimens from the São Paulo, Alambari, Brazil, were reared from Brazilian Peppertree (*Schinus terebinthifolius*). A specimen from Porto Feliz, São Paulo, Brazil, was reared from *Lithraea molleoides* (Vell.) Engl., Anacardiaceae.

#### Remarks.

*Paectes sinuosa* is described to differentiate the several species previously identified as *Paectes obrotunda* in the USNM collection.

*Paectes sinuosa* has two forms similar to those in *Paectes nana*. One form has bold markings of the antemedial and postmedial lines; the other form has faint antemedial and postmedial lines and an overall gray forewing. The male genitalia are identical in these two forms.

### Paectes tumida

Pogue sp. n.

urn:lsid:zoobank.org:act:6CF23F77-1A5E-46D6-A659-773A337E63B2

http://species-id.net/wiki/Paectes_tumida

Figs 26–28 36 43 49

#### Type material.

**Holotype** male – **SURINAME:** Geldersland, Collection Wm. Schaus, USNM ENT 00148679, genitalia slide male, USNM 135917 [green label]; HOLOTYPE / *Paectes tumida* Pogue” [red label]. Paratypes – (6 males, 4 females) **COLOMBIA:** META: Villavicencio, 400 m, (2 males), genitalia slides MGP 1299, MGP 1344, Fassl [BMNH]. **FRENCH GUIANA:** Nouveau Chantier, May (1 male), genitalia slide MGP 1308, Le Moult [BMNH]; St. Jean du Maroni, (1 male), genitalia MGP 1346, Le Moult [BMNH]. **GUYANA:** CUYUNI-MAZARUNI: Bartica, June 1901 (3 females), genitalia slide MGP 1307 [BMNH]; POTARO-SIPARUNI: Potaro River, 9-13 July 1912 (1male), genitalia slide MGP 1306, P. Rendall [BMNH]; Tumatumari, Dec. 1907 (1 female) [BMNH]. **SURINAME:** PARAMARIBO: Paramaribo, (1 male), Genitalia MGP 1347 [BMNH].

#### Etymology.

The species name is derived from the Latin *tumeo* (swell), referring to the swollen base of the free saccular extension in the male genitalia.

#### Diagnosis.

Forewing with a white medial area in *Paectes tumida*; in *Paectes similis* it consists of white-tipped gray scales, so the area is less contrasting than in *Paectes tumida*; in *Paectes obrotunda* medial area is mixed with gray, ferruginous, and a few cream-colored scales and also is less contrasting than in *Paectes tumida*. The postmedial line is faint and black in *Paectes tumida*; in *Paectes similis* it is black and well developed with a faint double line at the posterior margin; in *Paectes obrotunda* the postmedial line is ferruginous and double at the posterior margin and black and ferruginous where it curves toward the subapical black dash.

The male genitalia of *Paectes tumida* have elongate, curved setae on the dorsal surface of the valve, whereas in *Paectes obrotunda* and *Paectes similis* the valve has shorter, hairlike setae. The costal margin is convex in *Paectes tumida*, but straight in *Paectes similis*, and slightly concave in *Paectes obrotunda*. The free saccular extension is elongate and extends above the costa in *Paectes obrotunda*, but is shorter and does not extend above the costa in *Paectes tumida* and *Paectes similis*. In *Paectes tumida*, the base of free saccular extension is swollen and densely covered with spicules, but in *Paectes obrotunda* and *Paectes similis* the base is not swollen and without spicules.

#### Description.

**Male.** *Head* – antenna broadly bipectinate to 1/2 length then filiform; eyes large, globular; vertex with broad scales, mixture of white, brown, and black scales; frons with broad scales, projecting slightly beyond anterior eye margin, white and brown scales; labial palp porrect, mixture of brown, white, and pale ferruginous scales, internal surface white. *Thorax* – prothorax mixture of pale-gray, gray, and brown scales, medial band dark brown; patagium of brown, pale-ferruginous, and white broad scales, mixed with hairlike scales; protibia white mixed with black, apical band white, obscure, tarsi black with distinct white apical bands; middle and hind legs missing in holotype; forewing length 10.9 mm; costal area brown; basal area mixture of white, pale-ferruginous, and brown scales, not a well-defined ovate spot; antemedial line consists of a few black scales between posterior margin and anal vein then extending along anal vein a short distance before slightly curving upward; central area of wing from basal area to postmedial line mostly white mixed with brown scales and contrasted with subterminal and terminal areas, thin curved brown line from posterior margin to anal vein and contiguous with thin line from anal vein to CuA2 vein; reniform spot obscure, a pair of small, round brown spots; postmedial line brown and pale ferruginous, double line from posterior margin to vein CuA2, single curved line between M2 and M1; black dash between veins R5 and M1 that extends to outer margin; apical spot white; subterminal area brown mixed with pale ferruginous and white scales; terminal line a series of black, shallow scalloped lines between veins; fringe brown; hind wing marginal shading dark gray, veins heavily highlighted dark gray, areas between veins white; anal fold white with dark-gray striped pattern; fringe white. *Abdomen* –male eighth segment membranous with a pair of short, sternal, sclerotized bars and a pair of longer, wider, dorsal sclerotized bars; a pair of lateral, coremata bearing numerous, fine, elongate setae. *Genitalia* (Fig. 36) – Uncus triangulate, apex recurved and pointed, 1.3 × length of subscaphium; subscaphium, triangulate, decurved, apex pointed; valve membranous, short, truncate, covered with many elongate, curved setae; costa of valve extends above membranous part of valve, margin straight, apex broadly rounded, tuft of elongate setae near distal tip of apex; sacculus well developed, proximal half fused with valve, distal half free, sinuate, produced distally, apex round, covered with minute spicules; saccus triangulate; aedeagus straight, dorsum in distal third covered with minute spicules; base of vesica a short tube with two flat, elongate cornuti with pointed apices directed posteriorly, emerging from tubelike base, vesica ovate, apex of vesica with an irregular sclerotized area bearing a short, thumblike cornutus. **Female.** As in male except:antenna filiform; forewing length 11.5 mm; antemedial line dark brownish gray, faint, angulate from R vein to Cu vein then curved becoming a prominent black partial curve just dorsal to anal vein then straight to posterior margin; medial area less white than male; medial line dark brownish gray, series of variously sized, coalesced excurved lines from R vein to posterior margin; postmedial line dark brownish gray, double, slightly sinuate and fades medially. *Genitalia* (Fig. 43) – Papillae anales crescent shaped, soft, fleshy, covered with numerous setae; anterior apophyses fused with eighth sternite; posterior apophyses present; venter of eighth segment spiculate in distal half, sharply pointed projections laterally, small crescent-shaped, spiculate medial line; ostium bursae ovate with medial, curved, sclerotized bar; ductus bursae striate; appendix bursae juncture approximately midway between ostium bursae and corpus bursae, striate, curved; corpus bursae somewhat triangulate, covered internally with numerous thornlike signa pointing inward.

#### Distribution.

Specimens have been collected from Villavicencio, Colombia, and from Guyana, Suriname, and French Guiana (Fig. 49).

#### Remarks.

*Paectes tumida* is described to differentiate the several species previously identified as *Paectes obrotunda* in the USNM collection.

### Paectes obrotunda

(Guenée, 1852)

http://species-id.net/wiki/Paectes_obrotunda

Figs 44–47

Ingura obrotunda Guenée *in*Boisduval and Guenée 1852: 312.
Paectes obrotunda ; Hampson 1912: 130, pl. CLXXVII, Fig. 11; Franclemont and Todd 1983: 131; Poole 1989: 758; Poole and Gentili 1996: 762; Lafontaine and Schmidt 2010: 40.

#### Type material.

Type locality: “Brazil” Holotype female. BMNH; photographs of adult and genitalia examined (Figs 44–47).

#### Diagnosis.

Since *Paectes obrotunda* is only known from the female holotype it can be compared to females of *Paectes longiformis*, *Paectes sinuosa*, and *Paectes medialba*, which are found in Brazil, Argentina, and Paraguay. *Paectes obrotunda* has a distinct antemedial line that extends from the posterior margin to the anal vein then curves around faint basal area toward Cu vein. In *Paectes longiformis* the antemedial vein is faint and not well developed. The forewing subapical, marginal dash is black and distinct in *Paectes obrotunda*, but ferruginous and faint in *Paectes longiformis*. There is a faint area of white scales proximal to the postmedial line in *Paectes obrotunda*, which is absent in *Paectes longiformis. Paectes sinuosa* is distinct from *Paectes obrotunda* in having the antemedial line heavily developed with black scales along the anal vein and suffused with black scales along the posterior margin. The forewing subapical, marginal dash is longer in *Paectes sinuosa* than in *Paectes obrotunda*. In *Paectes medialba* the postmedial line is double from posterior margin to approximately middle of forewing whereas in *Paectes obrotunda* it is single and faint. The forewing subapical, marginal dash is slightly longer and more robust in *Paectes medialba* than in *Paectes obrotunda*. In the female genitalia the eighth sternite is longer than wide with lateral margins produced in *Paectes medialba* but sternite in *Paectes obrotunda* is wider than long and the lateral margins are not produced.

#### Redescription.

**Adult.** **Female.** *Head* – antenna filiform; eyes large, globular; labial palp porrect. *Thorax* – prothorax light brown, anterior margin thin black line; forewing length 9.2 mm; a few white scales forming short dashes along costa, especially from postmedial band to just below apex; thin black antemedial line from posterior margin forming ventral border to faint basal spot; medial area of wing with some scattered white scales forming an indistinct area proximal to postmedial line; postmedial line black, faint at posterior margin becoming more distinct prior to subapical, marginal dash; black dash between veins R5 and M1 that extends to outer margin; apical spot white; terminal line a series of black, recurved lines between veins; fringe with white patches giving a checked appearance; hind wing with dark gray marginal band that extends to middle of wing with veins highlighted dark gray. *Genitalia* (Fig. 46) – Papillae anales crescent shaped, soft, fleshy, covered with numerous setae; ninth sternite probably covered with minute spicules; eighth sternite with no distal apical lateral projections, lateral margins straight; ostium bursae somewhat sclerotized, band-like with lateral apices narrowed; base of ductus bursae narrower than ostium bursae; remainder of genitalia unknown.

**Figures 29–30.**
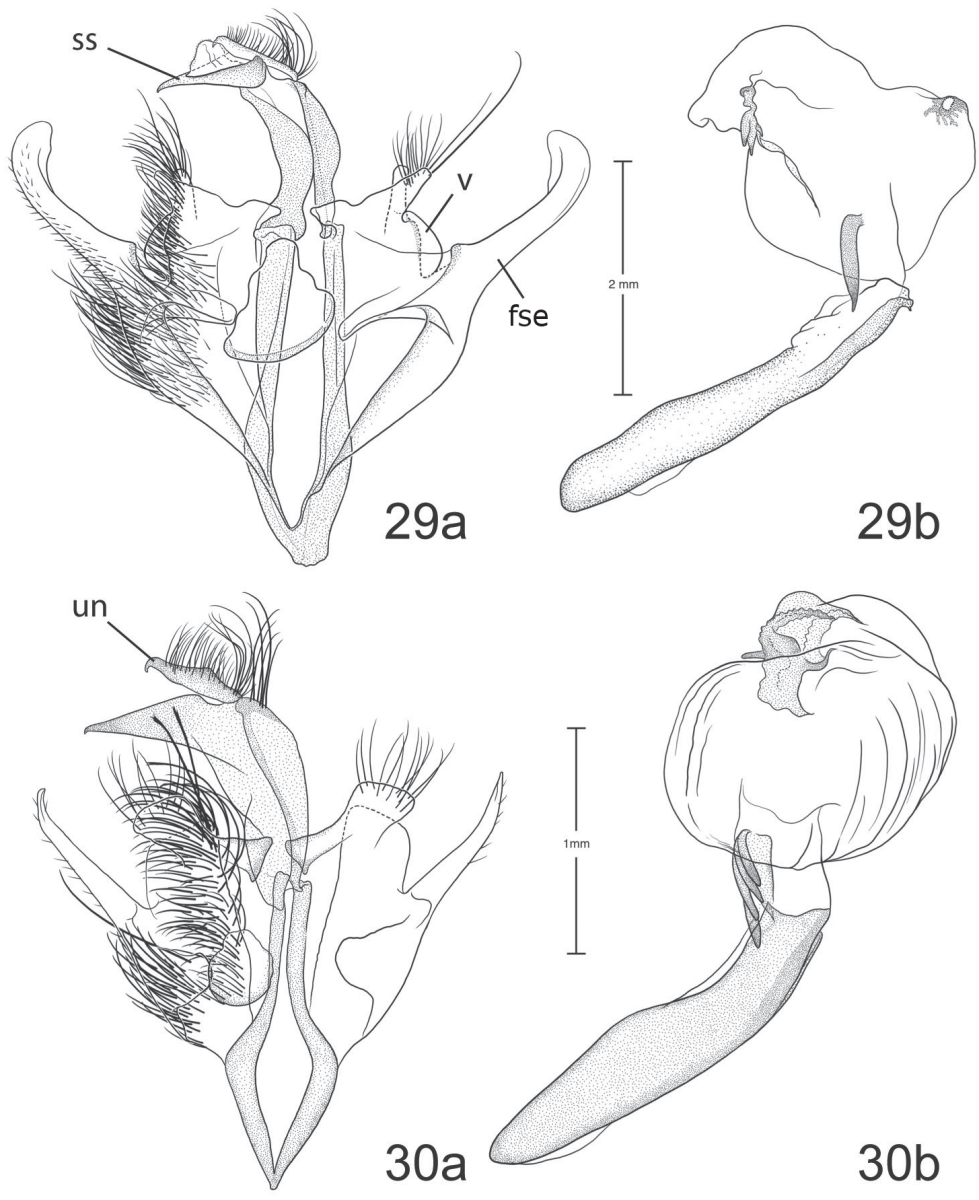
*Paectes* male genitalia; **fse** free saccular extension **ss** subscaphium **v** valve **un** uncus **29** *Paectes arcigera*.

**Figures 31–32.**
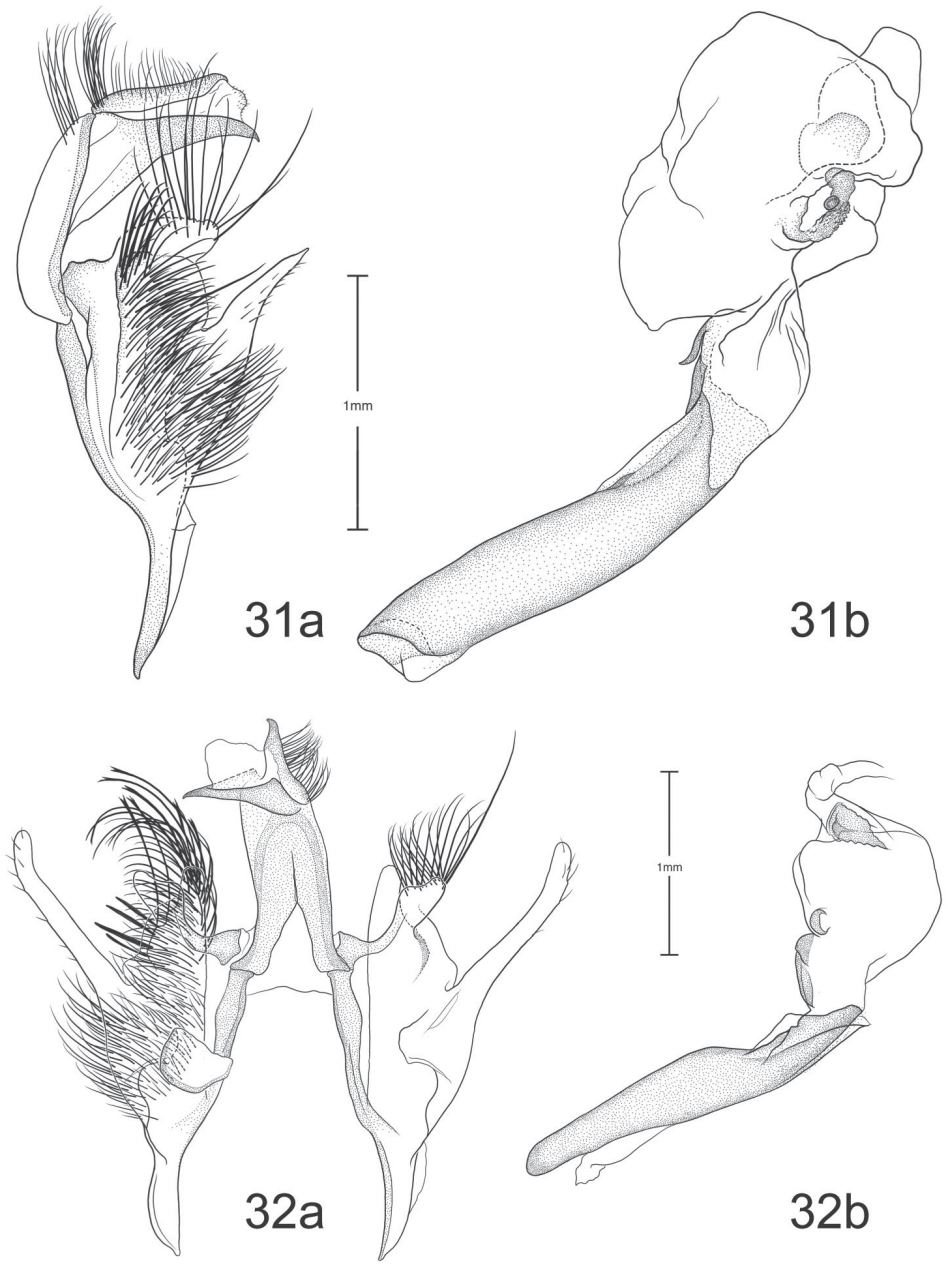
*Paectes* male genitalia. **31** *Paectes similis* **32** *Paectes nana*.

**Figures 33–34.**
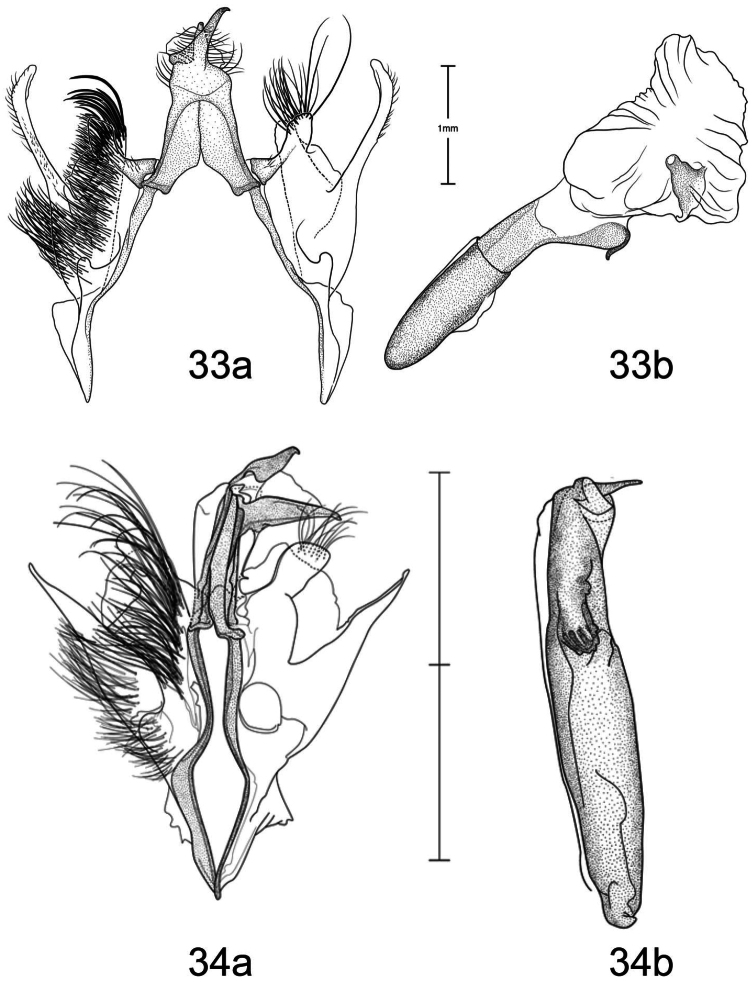
*Paectes* male genitalia. **33** *Paectes asper* **34** *Paectes medialba*.

**Figures 35–36.**
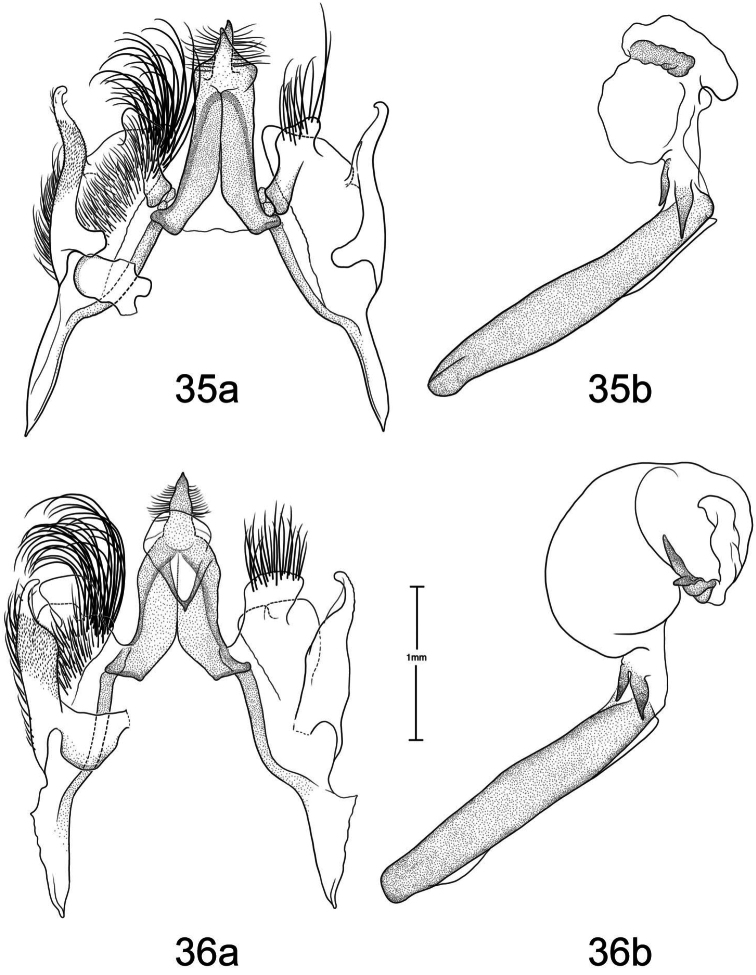
*Paectes* male genitalia. **35** *Paectes sinuosa* **36** *Paectes tumida*.

**Figures 37–38.**
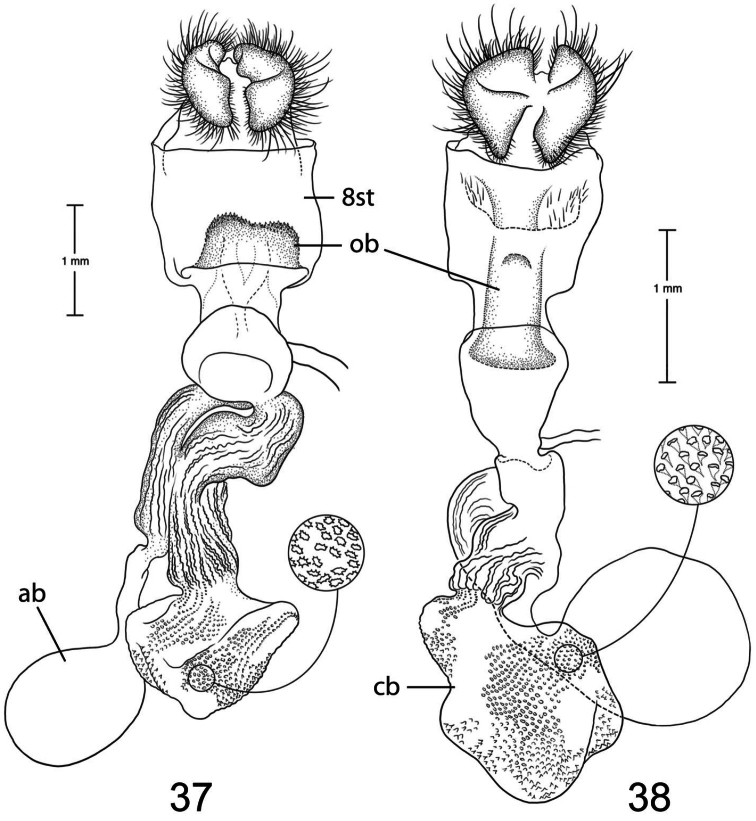
*Paectes* female genitalia; **8st** eighth sternite **ab** appendix bursae **ob** ostium bursae **cb** corpus bursae **37** *Paectes arcigera* **38** *Paectes longiformis*.

**Figures 39–40.**
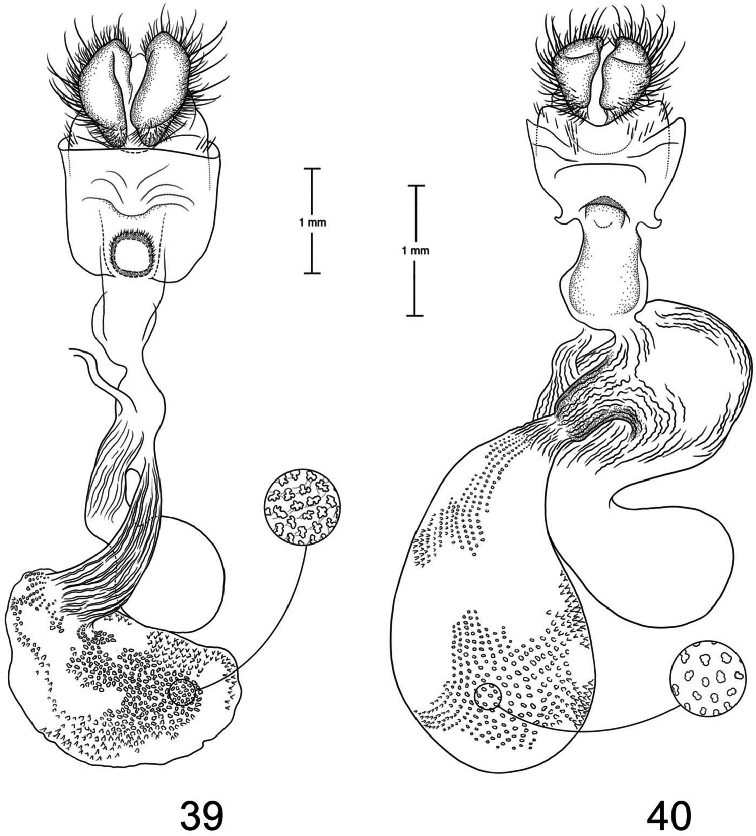
*Paectes* female genitalia. **39** *Paectes nana* **40** *Paectes asper*.

**Figures 41–42.**
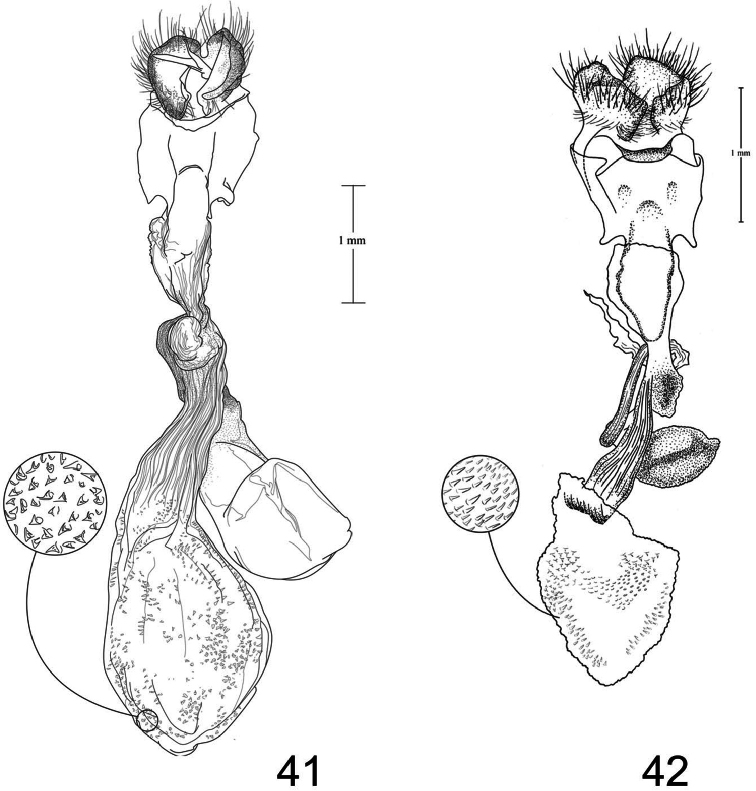
*Paectes* female genitalia. **41** *Paectes medialba* **42** *Paectes sinuosa*.

**Figure 43.**
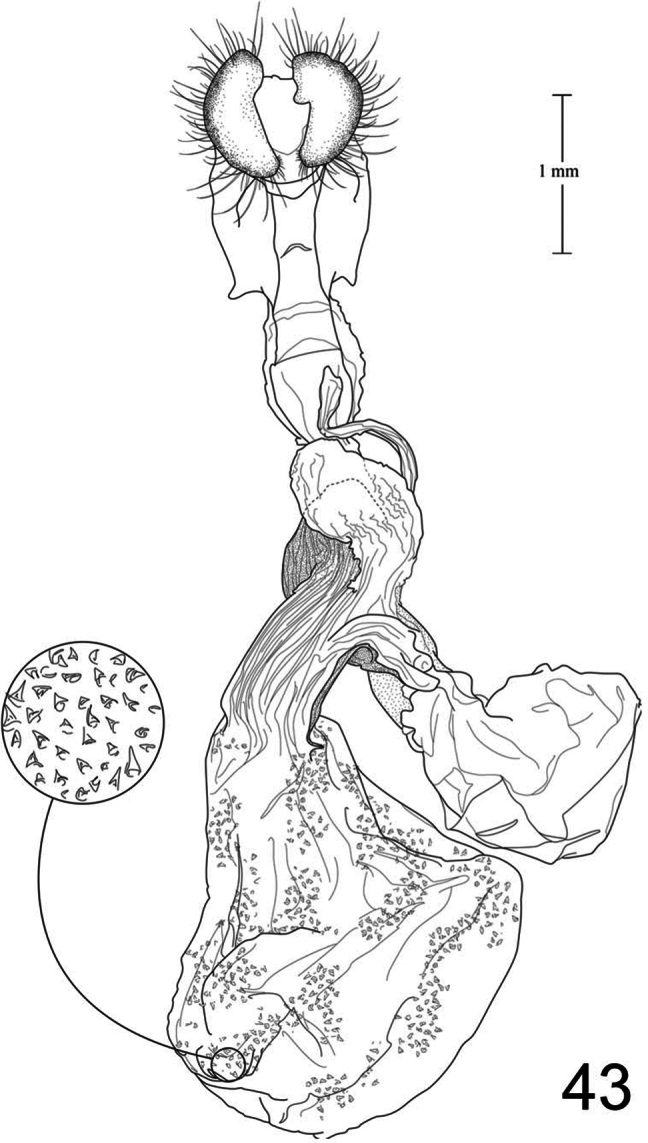
*Paectes tumida* female genitalia.

**Figures 44–47.**
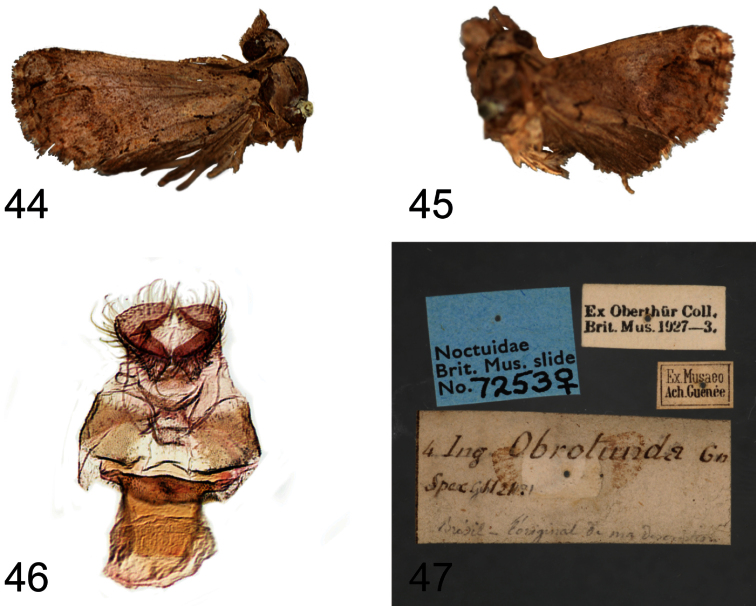
*Paectes obrotunda* (Guenée) female Holotype. **44** left forewing **45** right forewing **46 **genitalia **47** labels.

#### Distribution.

Known only from Brazil, with no specific locality.

**Figures 48–52.**
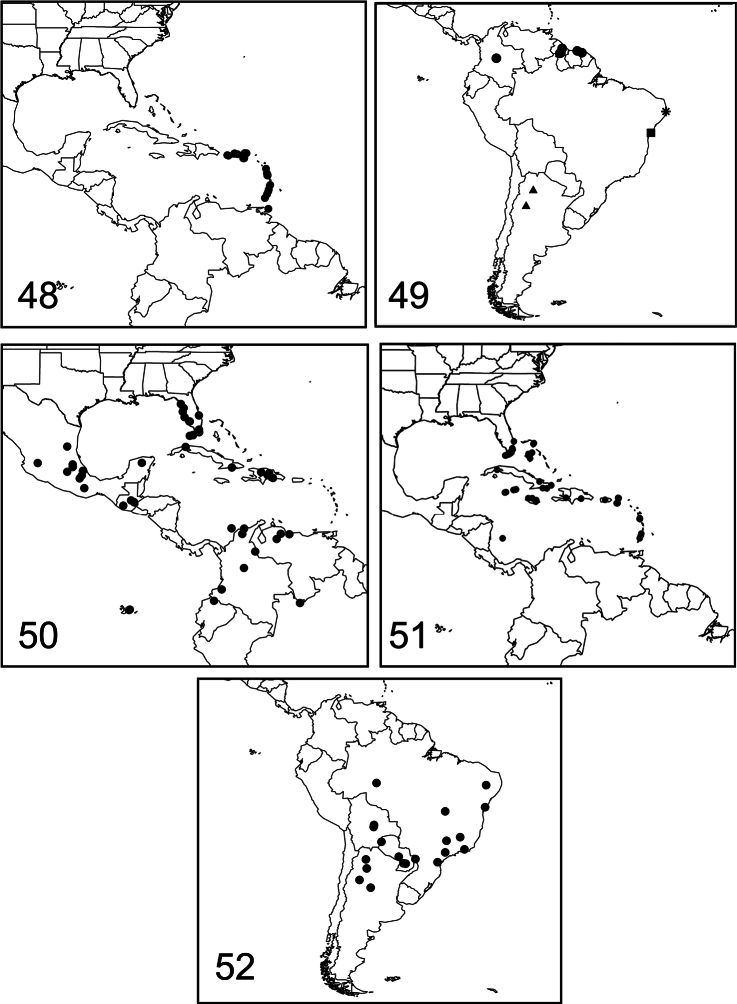
Distribution of collected specimens. **48 ***Paectes arcigera* **49** *Paectes longiformis* (square); *Paectes similis* (asterisk); *Paectes medialba* (triangle); *Paectes tumida* (circle) **50** *Paectes nana* **51** *Paectes asper* **52** *Paectes sinuosa*.

#### Remarks.

*Paectes obrotunda* belongs in this species group because it shares with them the shape of the antemedial line, white medial area, white apical spot, and black marginal dash in the forewing. The female genitalia are different from those of the other species in this group and no other specimens from other groups examined during this study matched them, so *Paectes obrotunda* is only known from the holotype.

*Paectes obrotunda* has never been correctly identified in the literature. Hampson (1912) gave a wide distribution of *Paectes obrotunda* extending from throughout the Caribbean to Paraguay. The Caribbean distribution could refer to either *Paectes asper*, *Paectes nana* or *Paectes arcigera*, and the Paraguay record is probably *Paectes sinuosa*. Kimball (1965) recorded *Paectes obrotunda* from Florida and these specimens can be referred to either *Paectes nana* or *Paectes asper*. Franclemont and Todd (1983) listed *Paectes obrotunda* from North America, undoubtedly following Kimball (1965).

Due to the cryptic nature of this species complex, the remaining species of Neotropical *Paectes* are currently being revised. A number of specimens from Costa Rica that have been analyzed using DNA barcoding of CO1 and their respective host plants will be included. A phylogenetic analysis using morphological characters of the Neotropical species included in this and the next study will be discussed.

## Supplementary Material

XML Treatment for Paectes arcigera

XML Treatment for Paectes longiformis

XML Treatment for Paectes similis

XML Treatment for Paectes nana

XML Treatment for Paectes asper

XML Treatment for Paectes medialba

XML Treatment for Paectes sinuosa

XML Treatment for Paectes tumida

XML Treatment for Paectes obrotunda
